# Machine learning–driven prediction of mechanical properties of lightweight concrete based on experimental data

**DOI:** 10.1038/s41598-026-55467-z

**Published:** 2026-06-03

**Authors:** Ahmed M. Gomaa, Mahmoud S. Ahmed, Ehab M. Lotfy, Erfan Abdel-Latif, Abdel-Rahman M. Naguib

**Affiliations:** 1 Construction and Building Engineering Department, Faculty of Engineering and Technology, Egyptian Chinese University, Cairo, Egypt; 2 Department of Structural Engineering, Faculty of Engineering, Suez Canal University, Ismailia, Egypt

**Keywords:** Lightweight concrete, Machine learning, Artificial neural network, Mechanical properties, Compressive strength, Tensile strength, Engineering, Materials science

## Abstract

Lightweight concrete (LWC) is increasingly used in structural and non-structural applications due to its ability to reduce self-weight while maintaining adequate mechanical performance. However, predicting the mechanical behavior of EPS-based lightweight concrete remains challenging because of the complex and nonlinear interactions between mix composition, density, curing age, and aggregate–matrix characteristics. This study proposes an integrated experimental and machine learning–based framework to predict the mechanical properties of lightweight concrete incorporating expanded polystyrene (EPS) particles (Addipor 55) as a partial volumetric replacement of natural coarse aggregate. An experimental program was conducted using EPS replacement levels ranging from 0 to 500 L/m³, combined with a fixed silica fume content of 60 kg/m³ and a polycarboxylate-based superplasticizer at a constant water–cement ratio of 0.35. Compressive strength, splitting tensile strength, and density were measured at curing ages of 3, 7, and 28 days. The results showed a systematic reduction in density from 2380 kg/m³ for the control mix to 1720 kg/m³ at the highest EPS content. The 28-day compressive strength decreased from 41.2 MPa to 28.6 MPa, while the splitting tensile strength declined from 4.30 MPa to 2.65 MPa. To enhance model robustness, the experimental dataset was expanded using a physically constrained data augmentation approach based on experimentally observed trends. An Artificial Neural Network (ANN) model was then developed and validated using independent data subsets, demonstrating excellent predictive performance with coefficients of determination (R²) of approximately 0.998 for both compressive and splitting tensile strengths. The proposed ANN model, supported by a graphical user interface (GUI), provides a reliable and efficient tool for predicting the mechanical performance of EPS-based lightweight concrete and optimizing mix design with reduced experimental effort.

**Supplementary Information:**

The online version contains supplementary material available at 10.1038/s41598-026-55467-z.

## Introduction

Concrete remains the most widely used construction material worldwide due to its versatility, durability, and relatively low cost. However, the increasing demand for concrete has raised significant environmental and structural concerns, particularly regarding the high self-weight of conventional concrete and the substantial carbon footprint associated with cement production^1,2^. The development of lightweight concrete (LWC) has therefore attracted growing attention as an effective solution to reduce structural dead loads, enhance seismic performance, and improve thermal insulation while maintaining adequate mechanical properties^3,4^.

Lightweight concrete can be produced using different approaches, including lightweight aggregates, cellular or foamed concrete, and the incorporation of polymer-based lightweight materials^5–7^. Among these approaches, the use of expanded polystyrene (EPS) particles as partial replacement of conventional aggregates has emerged as a promising technique due to the extremely low density, chemical stability, and availability of EPS materials^8^. EPS-based lightweight concrete exhibits reduced density and improved energy absorption capacity, making it particularly suitable for non-structural and semi-structural applications, as well as for seismic-resistant construction systems^9–12^.

Addipor 55 is a commercially available EPS-based granular material manufactured with controlled particle size distribution and low specific gravity. When incorporated into concrete mixtures, Addipor 55 acts as a lightweight aggregate substitute rather than an air-entraining agent^13,14^. The reduction in concrete density is therefore achieved through the inclusion of solid lightweight particles instead of entrained air voids. This distinction is critical, as EPS particles influence the mechanical behavior of concrete through particle–matrix interaction, stress redistribution, and modification of the interfacial transition zone (ITZ), rather than through the formation of air-void systems typically associated with air-entrained concrete^15^.

Despite the advantages of EPS-based lightweight concrete, the incorporation of EPS particles often leads to a reduction in compressive and tensile strengths due to the lower stiffness and strength of the polymeric inclusions compared with natural aggregates^16,17^. To mitigate this drawback, supplementary cementitious materials such as silica fume are commonly employed. Silica fume is characterized by its ultrafine particle size and high amorphous silica content, which significantly enhances pozzolanic reactivity and refines the microstructure of the cementitious matrix^18–21^. The combined use of EPS particles and silica fume can therefore provide a balanced lightweight concrete system with improved strength, durability, and interfacial bonding^22–24^. In addition, high-range water-reducing admixtures, such as polycarboxylate-based superplasticizers, are essential to maintain adequate workability at low water–cement ratios in mixtures containing silica fume and lightweight particles^25–27^.

The mechanical performance of EPS-based lightweight concrete is governed by a complex interaction among multiple parameters, including EPS content, density, binder composition, curing age, and water–cement ratio^28–30^. Traditional empirical or regression-based models often fail to accurately capture these nonlinear relationships. Advanced analytical and numerical models have been developed to represent nonlinear concrete behavior and confinement effects with higher accuracy, particularly under complex loading conditions^31–33^.

In recent years, machine learning (ML) techniques have emerged as powerful tools for modeling and predicting the behavior of complex cementitious systems. Among these techniques, Artificial Neural Networks (ANNs) have demonstrated strong capability in capturing nonlinear relationships between input variables and mechanical properties without requiring predefined formulations^34–36^.

Despite these advantages, existing studies on EPS-based lightweight concrete remain limited. Most research focuses primarily on compressive strength prediction using either experimental or data-driven approaches, often neglecting the combined prediction of multiple mechanical properties and the interaction between key governing parameters such as EPS content, density, and curing age.

Recent advances in artificial intelligence have shifted research toward integrated and hybrid frameworks that combine experimental data, advanced algorithms, and interpretability techniques. Hybrid machine learning approaches, including ensemble and deep learning models, have demonstrated improved prediction accuracy and generalization capability compared to standalone methods^37,38^.

In addition, increasing attention has been given to model interpretability and uncertainty quantification. Techniques such as feature importance analysis and SHAP provide insight into the influence of governing parameters, while uncertainty analysis enhances the reliability of machine learning predictions^39,40^.

In addition to ANN, several machine learning techniques such as Random Forest (RF), Support Vector Regression (SVR), and Gradient Boosting Machines (GBM) have been successfully applied to predict concrete properties^35,41,42^. These models offer advantages in terms of robustness and interpretability; however, their performance depends on dataset characteristics and problem complexity.

Despite these developments, many existing studies still rely on isolated modeling approaches without integrating experimental validation, multi-parameter prediction, and practical implementation within a unified framework.

Therefore, the novelty of the present study lies in the development of an integrated experimental–machine learning framework for EPS-based lightweight concrete. The proposed approach combines controlled experimental data with ANN modeling to simultaneously predict compressive and splitting tensile strengths while accounting for multiple interacting parameters. In addition, a graphical user interface (GUI) is developed to provide a practical and efficient engineering tool.

## Experimental study

### Materials

**Fig. 1:**
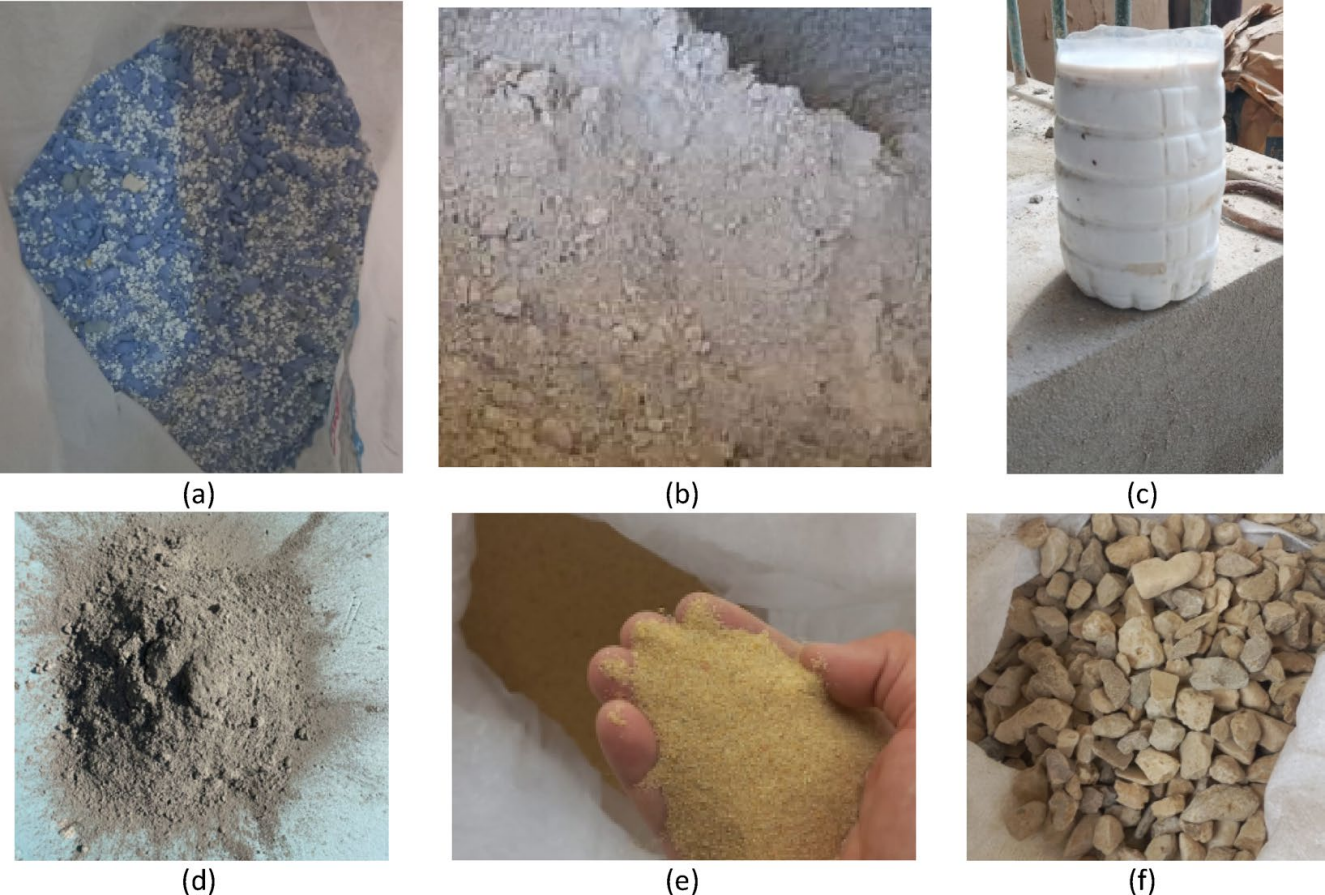
Materials used in the production of lightweight concrete: (**a**) expanded polystyrene (Addipor 55), (**b**) silica fume, (**c**) superplasticizer, (**d**) Ordinary Portland Cement, (**e**) natural fine aggregate, and (**f**) crushed coarse aggregate.

The materials used in this experimental study included Ordinary Portland Cement (OPC), natural fine aggregate (sand), natural coarse aggregate (crushed granite), expanded polystyrene (EPS) lightweight aggregate (Addipor 55), silica fume, a high-range water-reducing admixture (superplasticizer), and potable water. All materials were carefully selected to ensure consistency, repeatability, and compliance with relevant ASTM standards for concrete production. An overview of the materials employed in the experimental program is presented in Fig. 1.

Ordinary Portland Cement conforming to ASTM C150 Type I was used as the primary binder in all concrete mixtures. The cement exhibited stable hydration characteristics and consistent strength development, providing a reliable basis for evaluating the influence of EPS incorporation on the mechanical performance of lightweight concrete. The chemical composition of the cement was determined using X-ray fluorescence (XRF) analysis, and the results are summarized in Table S1.

Natural river sand complying with ASTM C33 was used as the fine aggregate. The sand was clean, well-graded, and free from deleterious materials. Crushed granite with a nominal maximum aggregate size of 20 mm was used as the coarse aggregate in the control mixture and was partially replaced in the lightweight concrete mixtures. The physical properties of both fine and coarse aggregates, including specific gravity, water absorption, bulk density, and gradation characteristics, were determined according to relevant ASTM standards and are presented in Table S2.

Lightweight concrete was produced using Addipor 55, a commercial expanded polystyrene (EPS) material supplied by CMB (Construction Materials Business). Addipor 55 consists of extruded EPS granules with controlled particle size and extremely low density, functioning as a lightweight aggregate rather than an air-entraining admixture. In this study, EPS particles were introduced as a partial volumetric replacement of natural coarse aggregate to achieve lightweight concrete characteristics through density reduction.

The physical and mechanical properties of the EPS particles, including particle size range, bulk density, specific gravity, water absorption, compressive resistance, and thermal conductivity, are summarized in Table S3. Due to its chemically inert nature, EPS does not participate in cement hydration reactions; instead, it modifies the composite behavior by reducing unit weight and altering stress transfer mechanisms within the concrete matrix.

Silica fume was incorporated as a supplementary cementitious material at a fixed dosage to enhance matrix densification and improve the interfacial transition zone between the cement paste and aggregates. A polycarboxylate-based superplasticizer (Viscocrete 3425) was used to maintain adequate workability, particularly in mixtures containing silica fume and EPS particles. Potable water suitable for drinking, in accordance with ASTM C1602, was used for both mixing and curing processes.

**Fig. 2:**
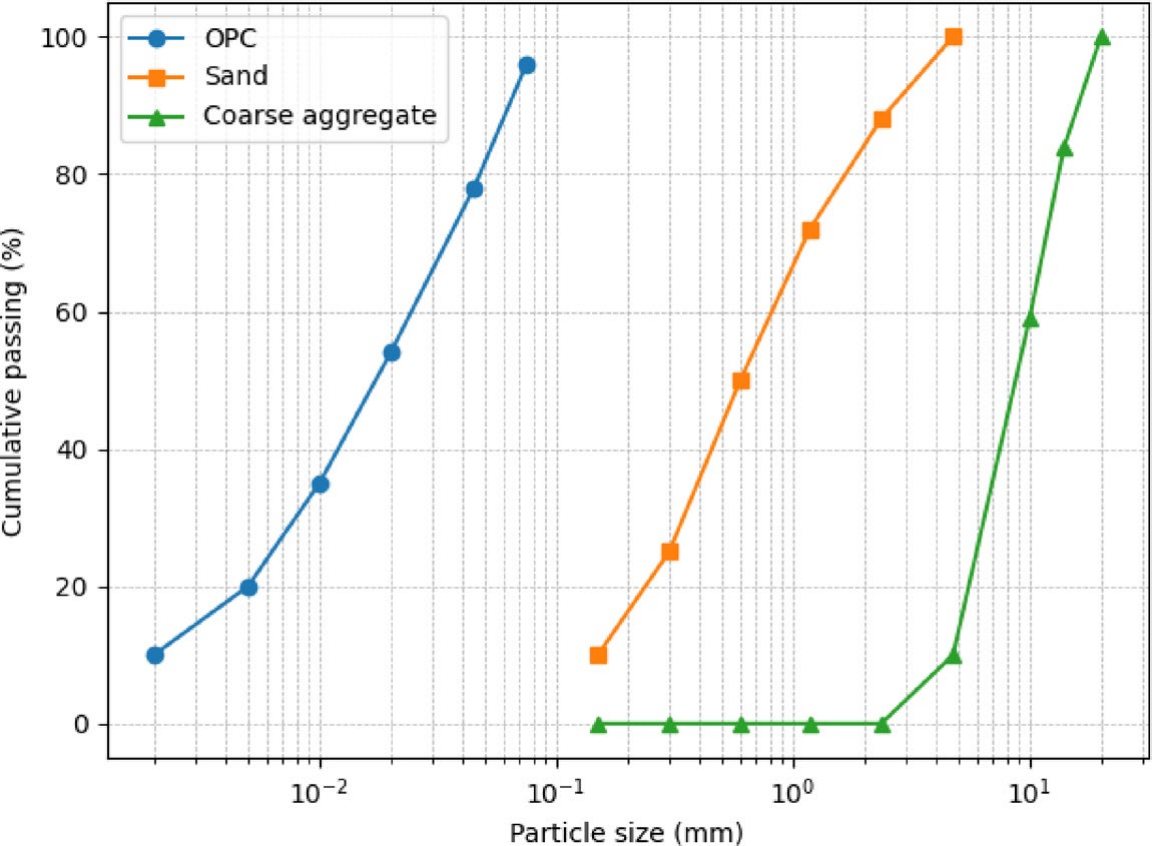
Particle size distribution (PSD) curves of Ordinary Portland Cement (OPC), natural fine aggregate, and coarse aggregate.

The particle size distribution (PSD) of OPC, fine aggregate, and coarse aggregate was evaluated to ensure proper grading and compatibility among the solid constituents. The PSD of the aggregates was determined experimentally using sieve analysis in accordance with ASTM C136, while cement PSD data were obtained from the manufacturer. The resulting PSD curves are presented in Fig. 2, confirming that the materials fall within acceptable grading limits and provide suitable particle packing for stable lightweight concrete mixtures.

### Mix design

The experimental program was designed to investigate the mechanical behavior of lightweight concrete produced by partial replacement of natural coarse aggregate with EPS (Addipor 55). A control mixture (C0) containing conventional aggregates and no EPS or silica fume was prepared as a reference. Additional mixtures incorporated silica fume at a constant dosage of 60 kg/m³ and a fixed superplasticizer content of 1.5% by weight of cement.

Lightweight concrete characteristics were achieved through the volumetric replacement of coarse aggregate with EPS particles, rather than through air entrainment. The EPS content was expressed as a foam-equivalent volume (L/m³), representing the volumetric fraction of EPS introduced into the mixture. As the EPS content increased, the mass of natural coarse aggregate was proportionally reduced to maintain an approximately constant total aggregate volume.

The water-to-cement ratio was maintained constant at 0.35 for all mixtures to isolate the effect of EPS replacement and binder modification on mechanical properties. The detailed mix proportions of all concrete mixtures are summarized in Table 1.

Preliminary trial mixes were conducted to ensure uniform dispersion of EPS particles, acceptable workability, and stable fresh concrete without segregation. This approach enabled systematic evaluation of density reduction and its influence on compressive and splitting tensile strengths, while generating a consistent dataset for subsequent machine learning modeling.

**Table 1:** Mix proportions of lightweight concrete mixtures.

Mix ID	Cement (kg/m³)	Fine Aggregate (kg/m³)	Coarse Aggregate (kg/m³)	EPS (Foam equivalent, L/m³)	w/c ratio	Silica fume (kg/m³)	Superplasticizer (Viscocrete 3425) (% of cement)
C0	400	650	**1200**	0	0.35	–	–
C-SF-0	340	650	**1200**	0	0.35	60	1.5
C-SF-10	340	650	**1080**	100	0.35	60	1.5
C-SF-20	340	650	**960**	200	0.35	60	1.5
C-SF-30	340	650	**840**	300	0.35	60	1.5
C-SF-40	340	650	**720**	400	0.35	60	1.5
C-SF-50	340	650	**600**	500	0.35	60	1.5

EPS (Foam equivalent) represents the volumetric equivalent of expanded polystyrene (EPS) particles used as partial replacement of coarse aggregate and does not indicate air entrainment.

### Specimen preparation

Concrete specimens were cast in standard steel molds and compacted using a vibrating table to ensure proper consolidation and uniform density. Special care was taken to avoid excessive vibration, which could cause segregation or floating of EPS particles. The fresh concrete was placed in molds in layers and compacted carefully to produce homogeneous specimens with consistent EPS distribution.

Cube specimens with dimensions of 150 mm × 150 mm × 150 mm were prepared for compressive strength testing, while cylindrical specimens with a diameter of 150 mm and a height of 300 mm were cast for splitting tensile strength evaluation. No flexural specimens were prepared, as the experimental program focused exclusively on compressive and tensile performance.

After casting, all specimens were covered to prevent moisture loss and demolded after 24 h. Subsequently, the specimens were cured in clean water at 20 ± 2 °C until the designated testing age.

For each concrete mixture, a total of six cube specimens and six cylindrical specimens were prepared. Two specimens were tested at each curing age (3, 7, and 28 days) for both compressive strength and splitting tensile strength tests to ensure consistency and repeatability of the experimental results. The reported compressive strength and splitting tensile strength values represent the average of the two tested specimens at each curing age.

### Testing procedures

Mechanical testing was conducted at curing ages of 3, 7, and 28 days in accordance with relevant ASTM standards. Compressive strength tests were performed on cube specimens following ASTM C39, while splitting tensile strength tests were conducted on cylindrical specimens in accordance with ASTM C496.

**Fig. 3:**
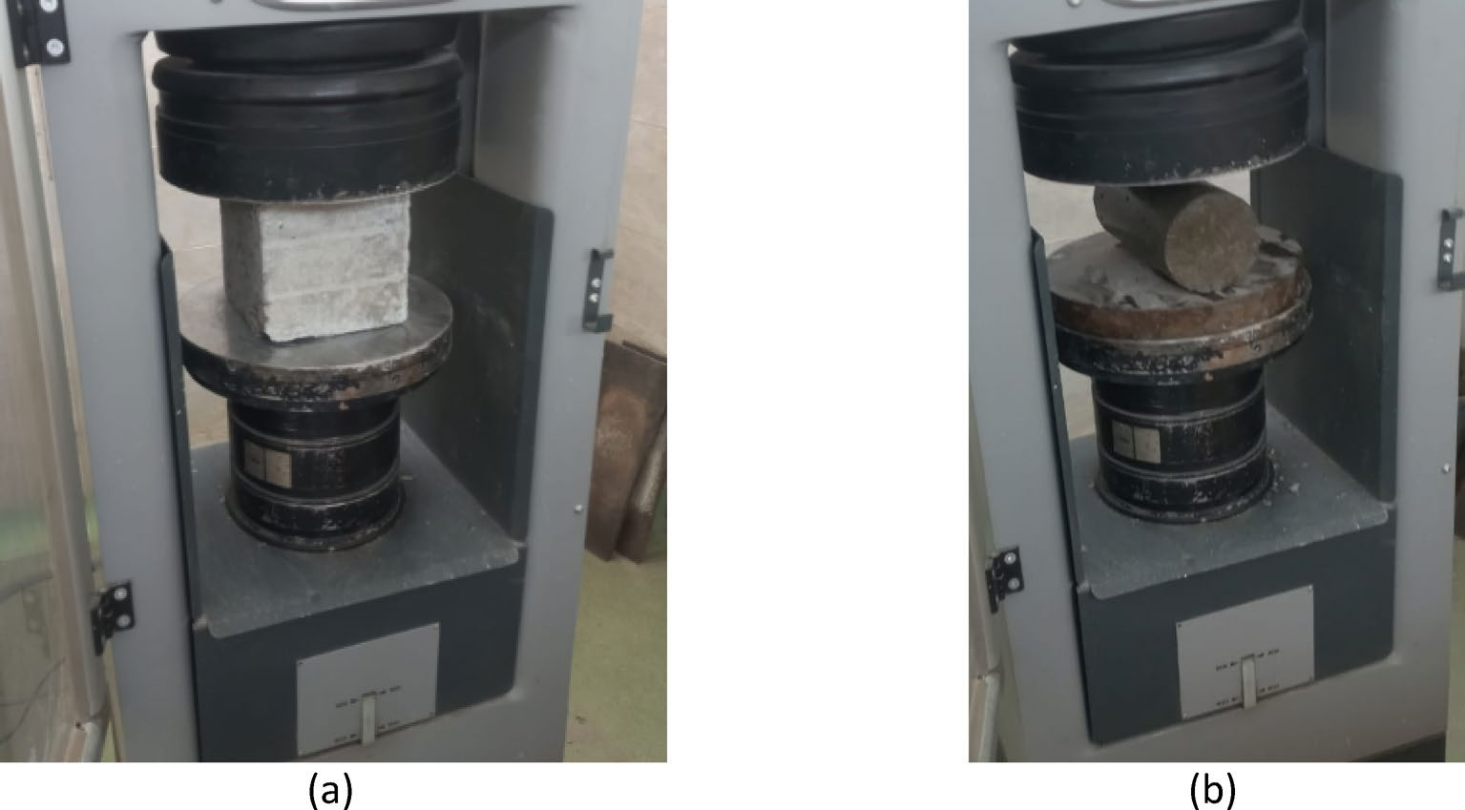
Testing setup and loading configurations for (**a**) compressive strength test on cube specimens and (**b**) splitting tensile strength test on cylindrical specimens.

All tests were carried out for the control mixture and the lightweight concrete mixtures containing different EPS replacement levels. In addition to strength testing, the hardened density of the concrete specimens was measured to quantify the effectiveness of EPS incorporation in producing lightweight concrete. The testing setup and loading configurations used for the mechanical tests are illustrated in Fig. 3.

### Results

The reported mechanical properties, including compressive strength and splitting tensile strength, correspond to the average values obtained from two specimens tested at each curing age. The variation between specimens was minimal, indicating good consistency and repeatability of the experimental results.

#### Compressive strength

**Fig. 4:**
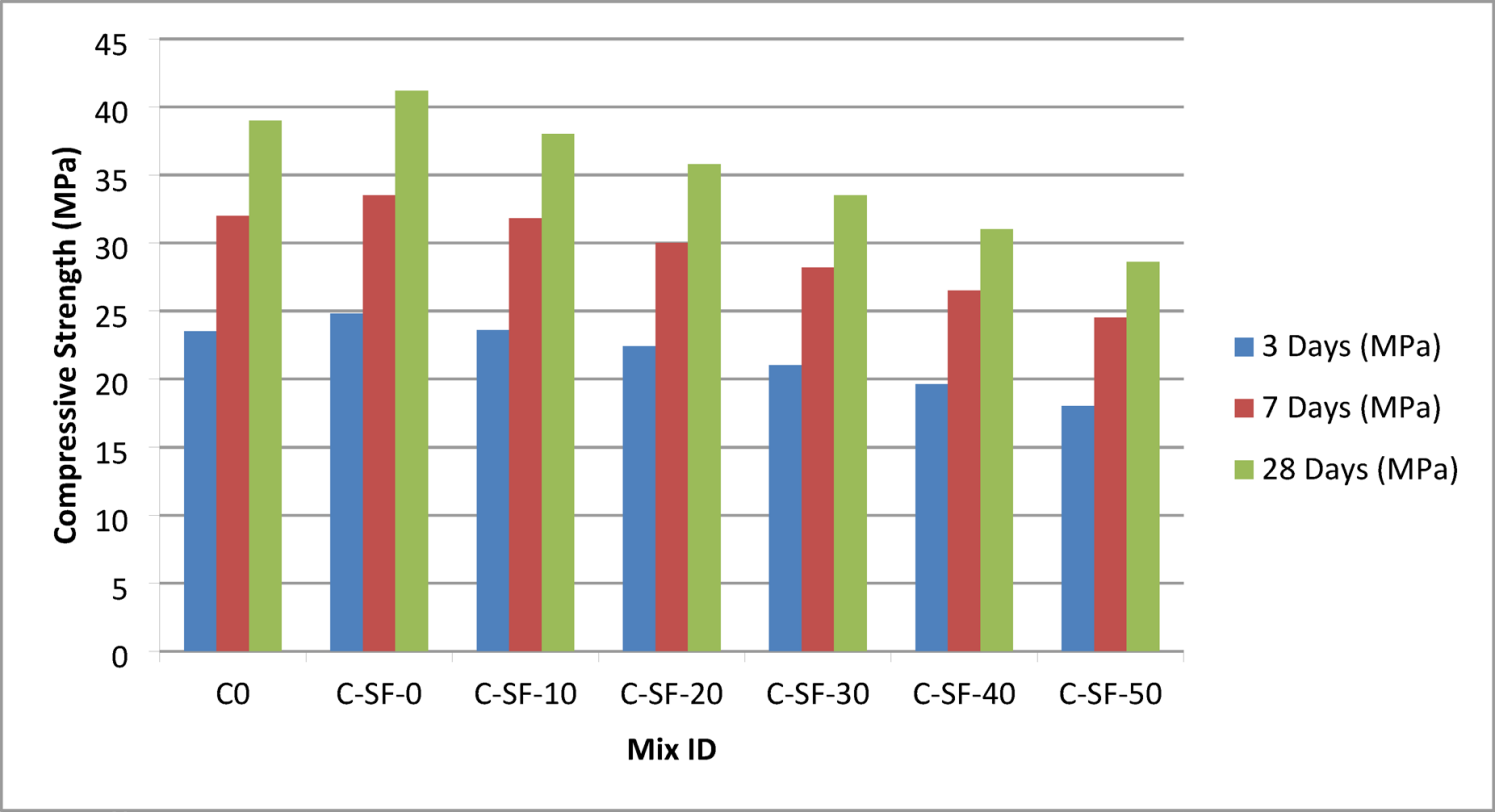
Variation of compressive strength of EPS-based lightweight concrete mixtures at 28 days.

The compressive strength results of the EPS-based lightweight concrete mixtures are summarized in Table S4 and illustrated in Fig. 4. The results clearly show that the reference mixture C0, produced with natural coarse aggregate and without EPS replacement, exhibited high compressive strength at all curing ages, reaching 39.0 MPa at 28 days. The incorporation of silica fume and superplasticizer in the mix C-SF-0, while maintaining zero EPS content, further enhanced the compressive strength, resulting in the highest recorded value of 41.2 MPa at 28 days. This improvement can be attributed to the pozzolanic activity of silica fume and its ability to refine the pore structure and strengthen the interfacial transition zone.

With the progressive introduction of EPS (Addipor 55) as a partial volumetric replacement of coarse aggregate in mixes C-SF-10 to C-SF-50, a gradual reduction in compressive strength was observed at 3, 7, and 28 days. As shown in Table S4 and Fig. 4, increasing the EPS content from 100 to 500 L/m³ led to a systematic decrease in compressive strength, with the 28-day strength reducing from 38.0 MPa for C-SF-10 to 28.6 MPa for C-SF-50. This reduction is primarily attributed to the replacement of stiff mineral coarse aggregates with lightweight EPS particles, which possess significantly lower stiffness, strength, and load-bearing capacity. Consequently, the continuity of stress-transfer paths within the concrete matrix is reduced, leading to lower resistance under compressive loading.

Despite the observed strength reduction, the decrease in compressive strength was not proportional to the increase in EPS content, indicating a nonlinear relationship between EPS volume and compressive performance. At moderate EPS replacement levels (100–200 L/m³), the loss in compressive strength remained relatively limited, demonstrating that a controlled substitution of coarse aggregate can achieve substantial density reduction while maintaining acceptable strength levels. This behavior highlights the beneficial role of the cementitious matrix enhanced with silica fume in partially compensating for the mechanical weakness introduced by EPS particles. The nonlinearity observed in Fig. 4 reflects the complex interaction between aggregate stiffness, matrix integrity, and density reduction, emphasizing the limitations of conventional empirical prediction models. Accordingly, these results provide strong justification for the application of machine learning techniques to accurately capture and predict the compressive strength behavior of EPS-based lightweight concrete.

#### Splitting tensile strength

**Fig. 5:**
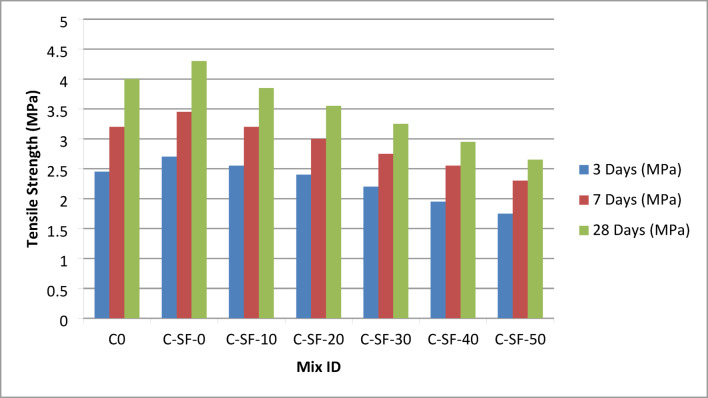
Variation of splitting tensile strength of EPS-based lightweight concrete mixtures at 28 days.

The splitting tensile strength results of the EPS-based lightweight concrete mixtures are presented in Table S5 and illustrated in Fig. 5. A trend consistent with that observed in compressive strength development was clearly identified at all curing ages. The reference mixture C0, containing natural coarse aggregate without EPS replacement, exhibited a relatively high splitting tensile strength, reaching 4.00 MPa at 28 days. The incorporation of silica fume and superplasticizer in mix C-SF-0, while maintaining zero EPS content, resulted in a further improvement in tensile performance, with the highest 28-day splitting tensile strength of 4.30 MPa. This enhancement is attributed to the refinement of the cementitious matrix and the improved bond between the cement paste and aggregates due to the pozzolanic activity of silica fume.

With the progressive replacement of coarse aggregate by EPS (Addipor 55) in mixes C-SF-10 to C-SF-50, a gradual and systematic reduction in splitting tensile strength was observed at 3, 7, and 28 days, as shown in Table S5 and Fig. 5. At 28 days, the splitting tensile strength decreased from 3.85 MPa for C-SF-10 to 2.65 MPa for C-SF-50 as the EPS content increased from 100 to 500 L/m³. This reduction is primarily attributed to the inherent mechanical weakness and low stiffness of EPS particles, which act as discontinuities within the concrete matrix and reduce the effectiveness of tensile stress transfer.

Splitting tensile strength is particularly sensitive to internal heterogeneity and weak inclusions. The presence of EPS particles introduces localized regions of reduced stiffness and poor crack-bridging capacity, which serve as preferential crack initiation points under tensile loading. As a result, tensile strength exhibited a more pronounced reduction compared to compressive strength at higher EPS contents. Nevertheless, the reduction in splitting tensile strength remained proportionally consistent with the corresponding reduction in compressive strength across all mixtures. This proportionality indicates stable and coherent mechanical behavior, despite the progressive increase in lightweight aggregate replacement.

The strong correlation observed between compressive and splitting tensile strengths confirms that both properties are governed by similar microstructural mechanisms, particularly matrix continuity, aggregate stiffness, and the quality of the interfacial transition zone. This consistency enhances the reliability of the experimental results and supports the suitability of the dataset as a robust target output for subsequent machine learning modeling aimed at predicting the mechanical performance of EPS-based lightweight concrete.

#### Relationship between density and mechanical properties

**Fig. 6:**
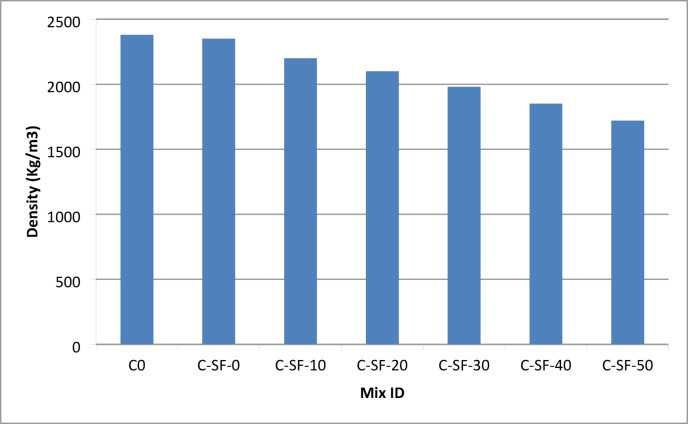
Relationship between concrete density and mechanical properties at 28 days.

The relationship between concrete density and mechanical performance of the EPS-based lightweight concrete mixtures is presented in Table S6 and illustrated in Fig. 6. The results clearly demonstrate a systematic reduction in concrete density with increasing EPS (Addipor 55) replacement levels, confirming the effectiveness of EPS as a lightweight aggregate substitute. The reference mix C0, containing natural coarse aggregate, exhibited the highest density of 2380 kg/m³, while the density gradually decreased to 1720 kg/m³ for mix C-SF-50, which incorporated the highest EPS content of 500 L/m³. A slight reduction in density was also observed between C0 and C-SF-0, despite both mixes having zero EPS content, which can be attributed to the partial replacement of cement with silica fume and the improved packing efficiency of the cementitious matrix.

Corresponding to the reduction in density, both compressive and splitting tensile strengths exhibited a decreasing trend, indicating a direct influence of density on mechanical performance. As EPS particles replaced dense mineral aggregates, the overall unit weight of concrete decreased, but this was accompanied by a reduction in stiffness and load-bearing capacity. The replacement of rigid coarse aggregates with lightweight EPS particles reduced the effectiveness of stress transfer within the concrete matrix, resulting in lower strength values at reduced density levels.

However, the relationship between density and mechanical properties was found to be nonlinear. As illustrated in Fig. 6, mixtures with similar density values exhibited noticeable variations in compressive and tensile strengths. This behavior indicates that density alone is insufficient to fully characterize the mechanical performance of EPS-based lightweight concrete. Factors such as EPS particle distribution, interfacial transition zone quality, aggregate–matrix bonding, and localized stress concentration effects play critical roles in governing strength behavior. In particular, the spatial distribution and volume fraction of EPS particles influence crack initiation and propagation mechanisms, leading to variations in strength even at comparable density levels.

The observed nonlinear density–strength relationship highlights the limitations of traditional empirical correlations that rely solely on density as a predictive parameter. Instead, it underscores the necessity of advanced data-driven approaches capable of capturing the complex interactions among density, mix composition, and mechanical response. This complexity provides strong justification for the application of machine learning techniques in the subsequent section, where multiple interacting variables are simultaneously considered to achieve accurate prediction of the mechanical properties of EPS-based lightweight concrete.

### Discussion

Compared to previous machine learning-based studies on concrete materials, the present work provides a more comprehensive framework by integrating experimentally derived data with a multi-parameter ANN model. Unlike conventional approaches that focus primarily on single-property prediction or rely on secondary datasets, this study simultaneously considers multiple governing variables and predicts both compressive and splitting tensile strengths within a unified model.

Recent advances in artificial intelligence for concrete materials have shifted toward integrated experimental–AI frameworks and hybrid modeling strategies^37,38^, where multiple algorithms, ensemble learning, and deep learning techniques are combined to improve predictive accuracy and robustness. These approaches have demonstrated superior performance compared to standalone models by capturing both linear and nonlinear relationships more effectively. In addition, modern studies increasingly emphasize model interpretability through feature importance analysis techniques, such as SHAP and sensitivity analysis, which provide insight into the relative influence of input parameters^43^. Similarly, uncertainty quantification has emerged as a critical aspect for evaluating the reliability and confidence of machine learning predictions in structural and materials engineering applications.

Within this context, the present study contributes by integrating controlled experimental data with ANN modeling to capture the complex interactions between EPS content, density, curing age, and mix composition. Although the adopted ANN model represents a single-model approach, it provides stable and physically consistent predictions supported by experimental validation. Furthermore, the developed graphical user interface enhances the practical applicability of the model, bridging the gap between advanced data-driven techniques and real-world engineering use. Nevertheless, extending the current framework toward hybrid modeling approaches, incorporating feature importance analysis, and integrating uncertainty quantification represents an important direction for future research to further enhance model interpretability and reliability.

#### Influence of EPS replacement on strength development with curing age

The experimental results demonstrate a clear and consistent increase in both compressive and splitting tensile strengths with curing age for all concrete mixtures, regardless of the EPS replacement level. This behavior reflects the progressive hydration of the cementitious matrix and the continued development of calcium silicate hydrate (C–S–H) gel, which enhances matrix densification and load transfer capacity over time. The incorporation of EPS particles did not interrupt the fundamental hydration mechanisms of Portland cement; however, it significantly influenced the rate and extent of strength development.

At early ages (3 days), the reduction in strength associated with increasing EPS content was more pronounced. At this stage, the cement matrix is still relatively immature, and the presence of EPS particles—characterized by low stiffness and weak interfacial bonding—limits the formation of a continuous load-bearing skeleton. As curing progressed to 7 and 28 days, continued hydration partially compensated for this effect, leading to improved strength development across all mixtures. Nevertheless, even at 28 days, mixes with higher EPS contents consistently exhibited lower strengths compared to the control mixtures, confirming that the mechanical contribution of EPS particles remains limited despite matrix maturation.

The presence of silica fume in EPS-containing mixtures further influenced strength development by refining the pore structure and improving the interfacial transition zone (ITZ) between the cement paste and aggregates. This effect explains the higher strength values observed for mix C-SF-0 compared to C0, despite both mixtures containing no EPS. The combined action of silica fume and superplasticizer resulted in enhanced particle packing and reduced capillary porosity, partially offsetting the negative effect of lightweight aggregate replacement.

#### Mechanisms governing compressive strength reduction

Compressive strength in concrete is primarily governed by the stiffness and continuity of the internal load transfer network formed by aggregates and cement paste. In EPS-based lightweight concrete, the progressive replacement of natural coarse aggregate with EPS particles leads to a fundamental alteration of this network. Unlike mineral aggregates, EPS particles possess extremely low elastic modulus and negligible compressive resistance, causing them to act as stress-relief inclusions rather than load-bearing constituents.

As EPS content increases, the effective load-bearing cross-sectional area within the concrete matrix decreases, resulting in localized stress concentrations around EPS particles under compressive loading. These stress concentrations promote microcrack initiation at lower stress levels, ultimately reducing the global compressive strength. The experimental results show that this reduction follows a nonlinear trend, particularly evident at higher EPS replacement levels. At moderate EPS contents (100–200 L/m³), the reduction in compressive strength remained relatively limited, suggesting that the cementitious matrix and remaining mineral aggregates were still capable of forming a sufficiently connected load path. However, beyond this threshold (300–500 L/m³), the cumulative loss of stiffness led to a more pronounced decline in strength.

This nonlinear behavior highlights that compressive strength reduction is not solely a function of EPS volume fraction, but also depends on EPS distribution, particle interaction, and matrix continuity. Such complexity cannot be adequately captured by linear empirical relationships, reinforcing the need for multivariate predictive models.

#### Sensitivity of splitting tensile strength to EPS incorporation

The splitting tensile strength exhibited trends similar to compressive strength but demonstrated a higher sensitivity to EPS replacement. Tensile failure in concrete is controlled by crack initiation and propagation, processes that are strongly influenced by internal heterogeneity and weak inclusions. The introduction of EPS particles creates localized zones of reduced stiffness and poor stress transfer capability, which act as preferential crack initiation sites under tensile loading.

As EPS content increased, the tensile strength reduction occurred at a slightly faster rate compared to compressive strength. This behavior is consistent with fracture mechanics principles, as tensile stresses are less tolerant of discontinuities within the matrix. Once a crack initiates at the EPS–paste interface, the low resistance of EPS particles provides minimal crack-bridging capacity, accelerating crack propagation through the matrix. Despite this sensitivity, the tensile-to-compressive strength ratio remained within ranges typically reported for lightweight concretes, indicating that the failure mechanism remained stable and did not shift toward brittle or anomalous behavior.

The strong correlation observed between compressive and splitting tensile strengths confirms that both properties are governed by similar microstructural mechanisms, primarily related to matrix integrity and aggregate stiffness. This consistency enhances the reliability of the experimental dataset and supports its suitability for use as target outputs in machine learning-based prediction models.

#### Ductility, failure behavior, and crack propagation

The incorporation of EPS particles significantly influences the failure behavior and ductility of lightweight concrete. Due to the low stiffness and low load-bearing capacity of EPS particles, the internal structure becomes more heterogeneous, which affects crack initiation and propagation mechanisms.

At higher EPS replacement levels, the presence of weak inclusions increases the likelihood of early crack initiation, particularly at the EPS–paste interface. These localized regions of reduced stiffness act as stress concentration points, which may promote more localized cracking and potentially lead to a more brittle response under loading conditions. However, EPS particles can also contribute to improved energy absorption capacity due to their deformability, which may partially enhance ductility at moderate replacement levels. This suggests that the mechanical behavior of EPS-based concrete is governed by a balance between reduced stiffness and increased deformation capacity.

Crack propagation in EPS-based concrete differs from conventional concrete, as cracks tend to develop around EPS particles and follow paths of least resistance through weak interfaces. This behavior influences both strength and post-peak response, although the latter was not directly evaluated in the present study. From a structural perspective, these characteristics have important implications under seismic or dynamic loading. Reduced stiffness and altered crack patterns may affect stiffness degradation, energy dissipation, and overall structural response. Therefore, careful consideration of EPS content is required when such materials are used in structural or seismic applications.

#### Density–strength relationship and lightweight concrete classification

The density results clearly demonstrate the effectiveness of EPS replacement in producing lightweight concrete. A systematic reduction in density was observed with increasing EPS content, transitioning the material from conventional concrete density levels to values commonly associated with structural and semi-structural lightweight concrete. However, the relationship between density and mechanical properties was found to be inherently nonlinear.

Although lower density generally corresponded to lower strength, mixtures with comparable densities exhibited noticeable variations in both compressive and tensile strengths. This behavior indicates that density alone is insufficient to fully characterize mechanical performance. Factors such as EPS particle size and distribution, interfacial bonding quality, cement matrix homogeneity, and stress redistribution mechanisms play decisive roles in determining strength at a given density level. Consequently, two mixtures with similar densities may exhibit different mechanical responses depending on their internal structure.

This finding has important practical implications. While density reduction is a primary objective in lightweight concrete design, achieving an optimal balance between weight reduction and mechanical performance requires careful control of mix composition and EPS content. The ability to achieve significant density reduction while maintaining acceptable strength levels highlights the practical viability of EPS-based lightweight concrete for applications where reduced self-weight is critical.

Although the present study focuses on strength-related properties, incorporating additional parameters such as elastic modulus and full stress–strain behavior would provide a more comprehensive understanding of the mechanical response of EPS-based lightweight concrete, particularly in relation to stiffness and ductility characteristics.

## Machine learning study

### Development of the ANN model

To develop an Artificial Neural Network (ANN) model capable of accurately predicting the mechanical properties of EPS-based lightweight concrete, a comprehensive and reliable dataset was required. The mechanical behavior of lightweight concrete incorporating expanded polystyrene (EPS) as a partial volumetric replacement of coarse aggregate is governed by complex and nonlinear interactions among mix composition, density, curing age, and internal heterogeneity introduced by EPS particles. Conventional empirical models are often inadequate in capturing these interactions; therefore, a data-driven modeling approach was adopted in this study to accurately represent the underlying physical behavior.

**Fig. 7:**
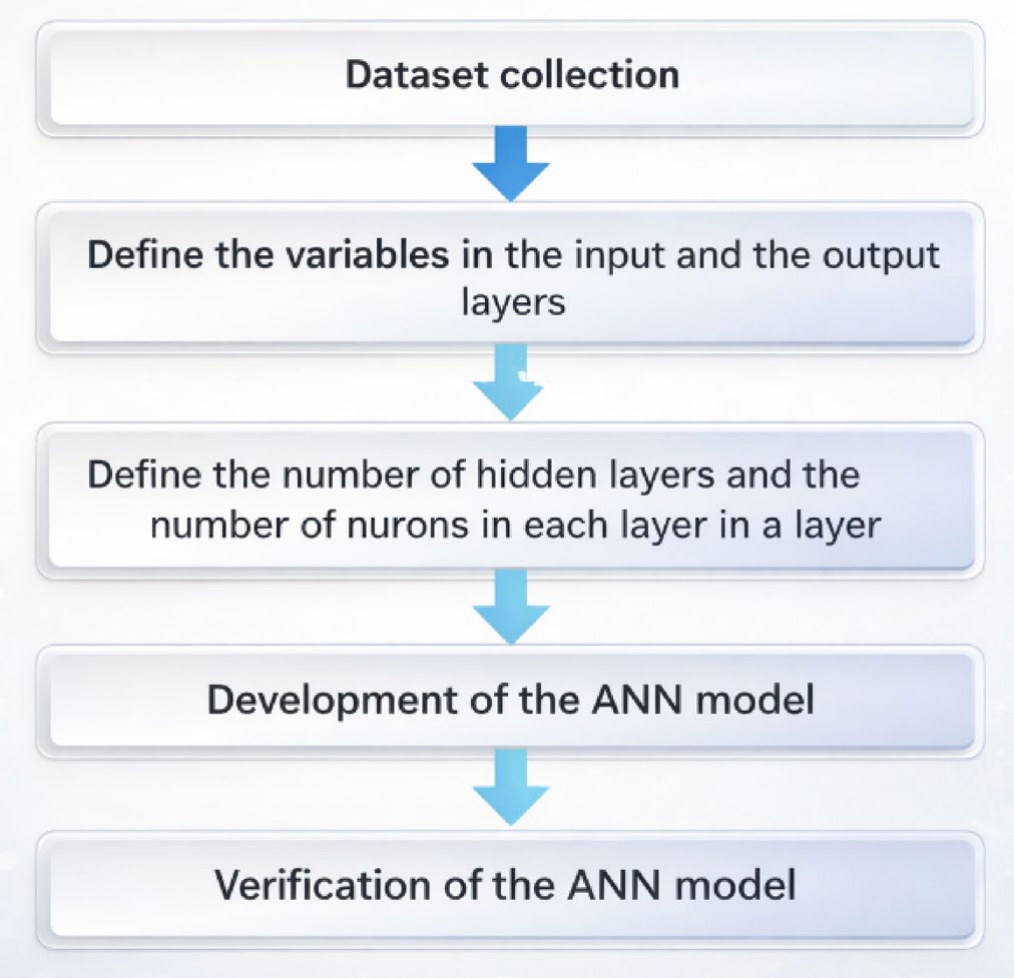
Simplified flow diagram for the proposed framework.

The adopted machine learning framework relied on experimentally measured data obtained from laboratory testing of EPS-based lightweight concrete mixtures with varying EPS contents. The dataset included compressive strength, splitting tensile strength, and density values measured at different curing ages. To enhance the robustness and generalization capability of the ANN model, the experimental dataset was augmented within the bounds defined by observed experimental trends. Several ANN architectures were examined by varying the number of neurons in the hidden layer, and the predictive performance of each configuration was evaluated using statistical indicators such as the coefficient of determination (R²), root mean squared error (RMSE), and mean absolute error (MAE). Based on these criteria, the ANN model exhibiting the best overall predictive performance was selected as the final model. Subsequently, a parametric study was conducted using the trained ANN to investigate the relative influence of key input parameters on the mechanical properties of EPS-based lightweight concrete. A simplified flow diagram illustrating the adopted machine learning framework is presented in Fig. 7.

### Dataset preparation

The accuracy and reliability of machine learning models strongly depend on the quality, consistency, and diversity of the training dataset. In the present study, the dataset was carefully prepared to represent the mechanical behavior of EPS-based lightweight concrete over a wide range of EPS replacement levels and corresponding density variations.

It is important to emphasize that the core dataset was primarily derived from controlled laboratory experiments conducted in this study. The experimental program was designed to systematically vary the EPS replacement level while maintaining controlled conditions for other key parameters, ensuring high data reliability, consistency, and reproducibility. All experimental measurements, including compressive strength, splitting tensile strength, and density, were obtained following relevant ASTM standards, which further enhances the credibility of the dataset.

Based on previous studies on lightweight concrete and polymer-modified aggregate systems, the selected input variables cement content, fine and coarse aggregate contents, water–cement ratio, EPS content, concrete density, and curing age represent the most influential parameters governing compressive and splitting tensile strengths. The dataset spans a wide range of EPS contents (0–500 L/m³), curing ages (3–28 days), and corresponding density variations, ensuring that the data are representative of practical engineering applications and typical lightweight concrete design conditions.

The complete dataset used for ANN modeling was obtained from two complementary sources: experimentally measured results obtained from laboratory testing, and dataset augmentation performed within the limits defined by experimentally observed behavior.

The experimental program resulted in a total of 21 data points obtained from compressive strength, splitting tensile strength, and density measurements at different curing ages. To improve the robustness and generalization capability of the ANN model, the dataset was expanded to a total of 188 samples through a controlled augmentation process.

The augmented data were generated strictly within the bounds of the experimental dataset and guided by physically meaningful relationships observed during testing. The final dataset, combining both experimental and augmented data, was used for ANN training and evaluation. All data are presented in Table S7.

#### Dataset from experimental testing

The experimental dataset was obtained from compressive and splitting tensile strength tests conducted on EPS-based lightweight concrete mixtures. Cube specimens with dimensions of 150 mm × 150 mm × 150 mm were used for compressive strength testing, while cylindrical specimens with dimensions of 150 mm × 300 mm were employed for splitting tensile strength evaluation. Mechanical tests were conducted at curing ages of 3,7, and 28 days to capture short, and long-term strength development.

The experimentally measured parameters included cement content, fine aggregate content, coarse aggregate content, water–cement ratio, EPS replacement level expressed as foam equivalent volume, concrete density, curing age, compressive strength, and splitting tensile strength. These experimental results formed the core dataset used for training, testing, and validating the ANN model.

#### Dataset augmentation

To overcome the inherent limitations associated with the size of purely experimental datasets and to improve the generalization capability of the ANN model, a controlled dataset augmentation strategy was employed. It is important to note that the augmentation process was not based on random data generation; rather, it was carefully guided by experimentally observed trends and well-established physical behavior of EPS-based lightweight concrete.

The original experimental dataset consisted of 21 data points obtained from laboratory measurements. To enhance data diversity and improve model robustness, the dataset was expanded to a total of 188 samples through a structured augmentation process.

Additional data points were generated by interpolating within the minimum and maximum bounds of the experimental dataset, ensuring that no extrapolation beyond physically meaningful ranges was introduced. This constraint guarantees that all augmented data remain consistent with realistic material behavior and prevents the inclusion of non-physical or misleading patterns.

To further ensure dataset credibility, several governing constraints were imposed during the augmentation process. These include: (i) the monotonic decrease in concrete density with increasing EPS replacement level, (ii) the progressive increase in compressive and splitting tensile strengths with curing age, and (iii) the nonlinear relationship between density and mechanical properties. These constraints were directly derived from experimental observations and supported by established findings in the literature.

Furthermore, the augmentation process was designed to preserve the statistical distribution and variability of the original dataset, ensuring balanced data coverage across the entire parameter space. The final combined dataset, consisting of both experimental and augmented data, was randomly divided into 70% for training, 15% for validation, and 15% for testing, ensuring representative data distribution across all subsets. By adhering to these physically informed constraints, the augmented dataset improves the diversity and generalization capability of the model without compromising data integrity or scientific reliability.

### Dataset description

The dataset employed for developing the Artificial Neural Network (ANN) model comprises a set of input and output variables that collectively describe the mechanical behavior of EPS-based lightweight concrete. The input variables constitute the neurons of the ANN input layer, while the corresponding mechanical properties form the output layer. A comprehensive statistical summary of all variables, including minimum, maximum, mean, and standard deviation values, is provided in Table 2. This statistical overview confirms that the dataset spans a sufficiently wide range of values, which is essential to ensure robust ANN training, enhance generalization capability, and achieve reliable prediction accuracy.

The ANN input variables represent the key parameters governing the performance of EPS-based lightweight concrete. These variables include cement content (kg/m³), fine aggregate content (kg/m³), coarse aggregate content (kg/m³), water–cement ratio, EPS content expressed as foam equivalent volume (L/m³), concrete density (kg/m³), and curing age (days). Collectively, these parameters capture the combined effects of mixture composition, internal pore structure induced by EPS inclusion, and hydration development over time on the resulting mechanical properties.

The output variables of the ANN model correspond to the primary mechanical properties investigated in this study, namely compressive strength (MPa) and splitting tensile strength (MPa). These properties were selected due to their fundamental role in evaluating structural performance and their proven sensitivity to variations in EPS content and concrete density.

**Fig. 8:**
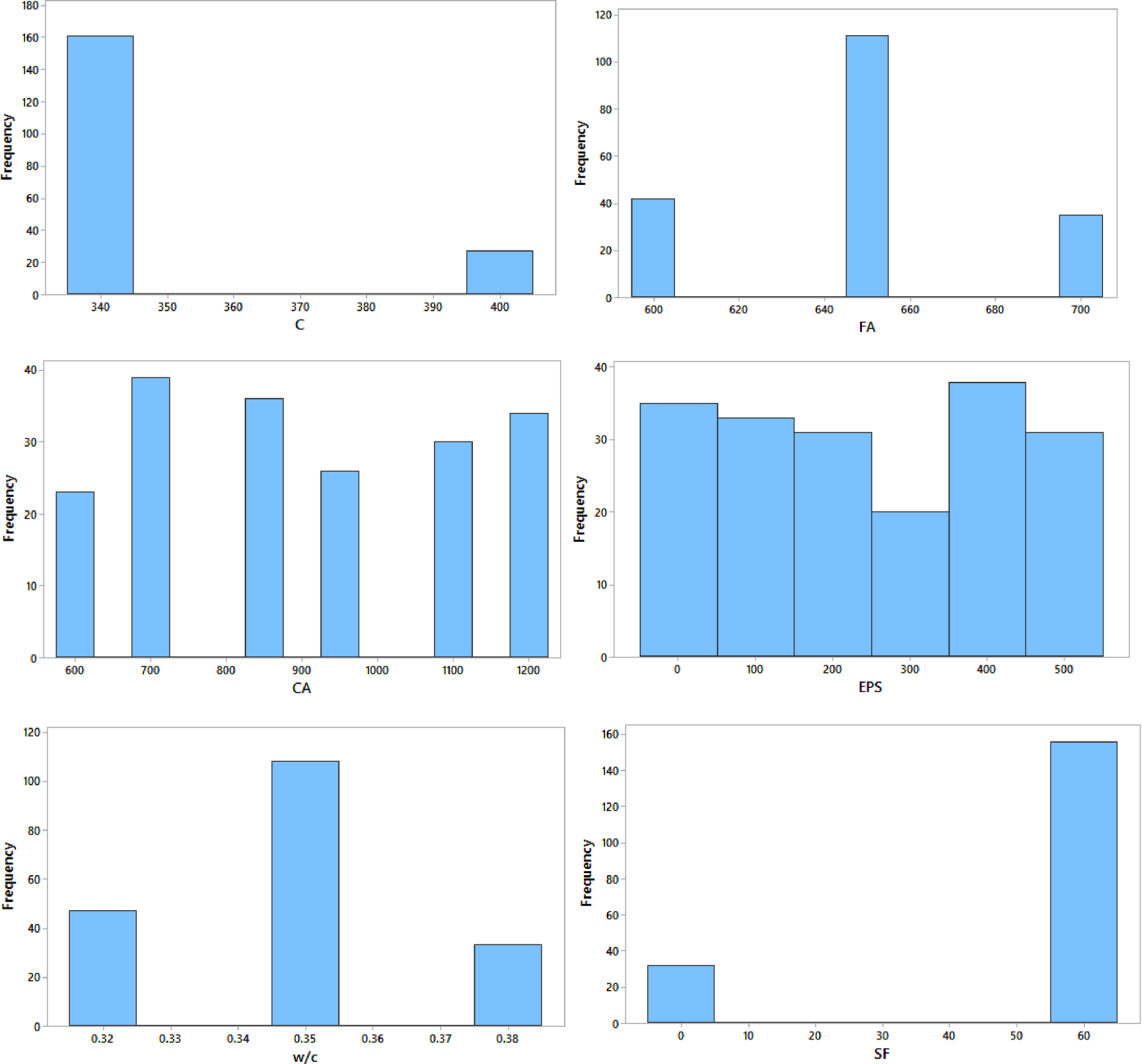

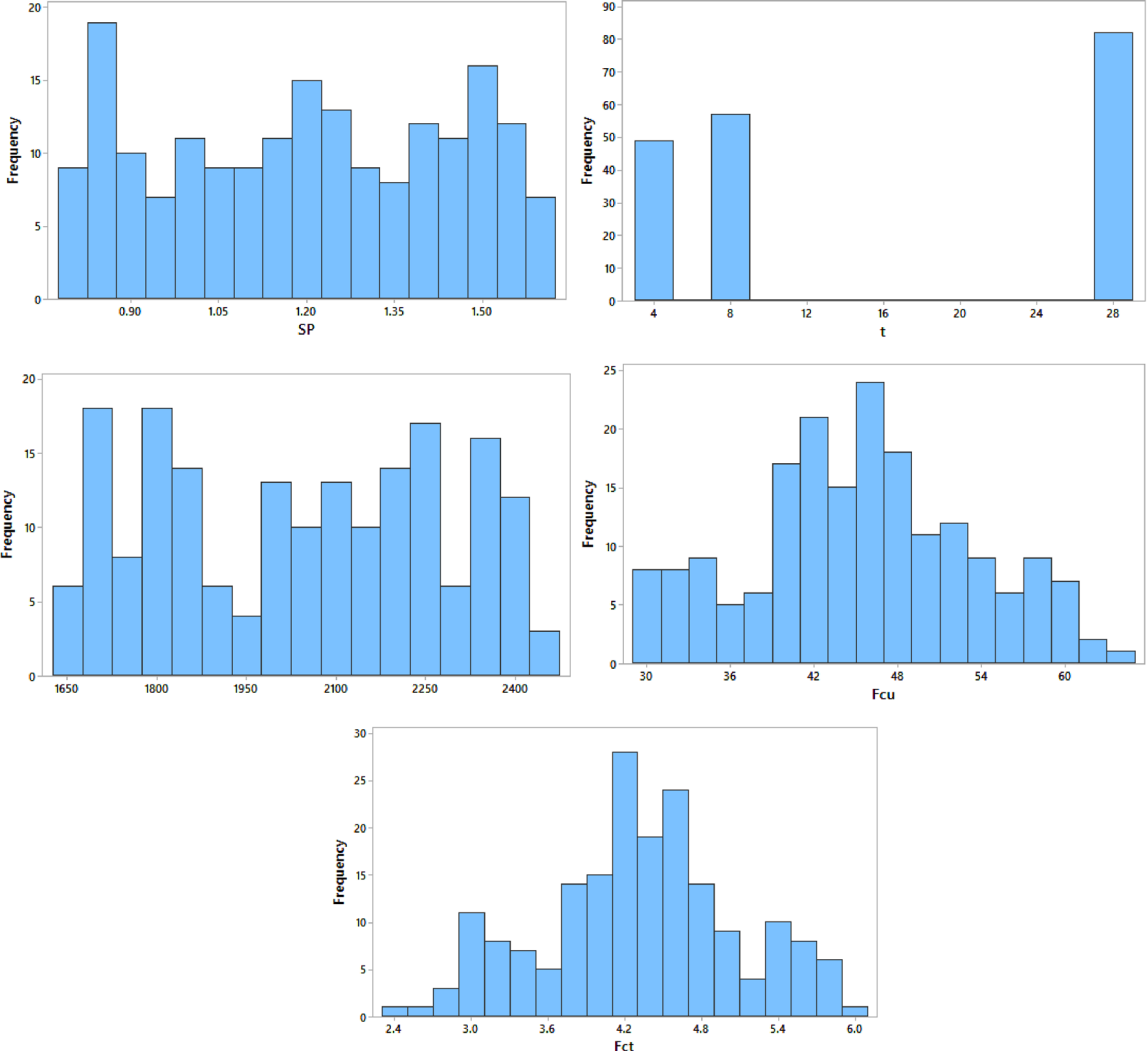
Frequency distribution of input, and output variables.

Furthermore, the frequency distributions of the input variables, illustrated in Fig. 8, demonstrate that the dataset provides adequate and uniform coverage across the investigated parameter ranges. The absence of significant data clustering or imbalance confirms the suitability of the dataset for ANN modeling and reduces the risk of biased training outcomes.

**Table 2:** Statistical description of input and output variables used for ANN modeling.

Variable	Symbol	Unit	Min	Max	Mean	Std. Dev.
Cement content	C	kg/m³	340	400	350.6	21.8
Fine aggregate content	FA	kg/m³	600	700	649.2	38.4
Coarse aggregate content	CA	kg/m³	600	1200	918.6	201.3
EPS content (foam equivalent)	EPS	L/m³	0	500	247.3	152.8
Water–cement ratio	w/c	–	0.32	0.38	0.35	0.02
Silica fume content	SF	kg/m³	0	60	47.8	22.1
Superplasticizer dosage	SP	% of cement	0.80	1.60	1.21	0.23
Curing age	t	days	3	28	15.6	10.2
Concrete density	ρc	kg/m³	1680	2390	2035.4	184.6
Compressive strength	F_cu_	MPa	18.2	42.8	31.4	6.1
Splitting tensile strength	F_ct_	MPa	1.65	4.25	2.98	0.62

### Effective parameters and correlation analysis

**Fig. 9:**
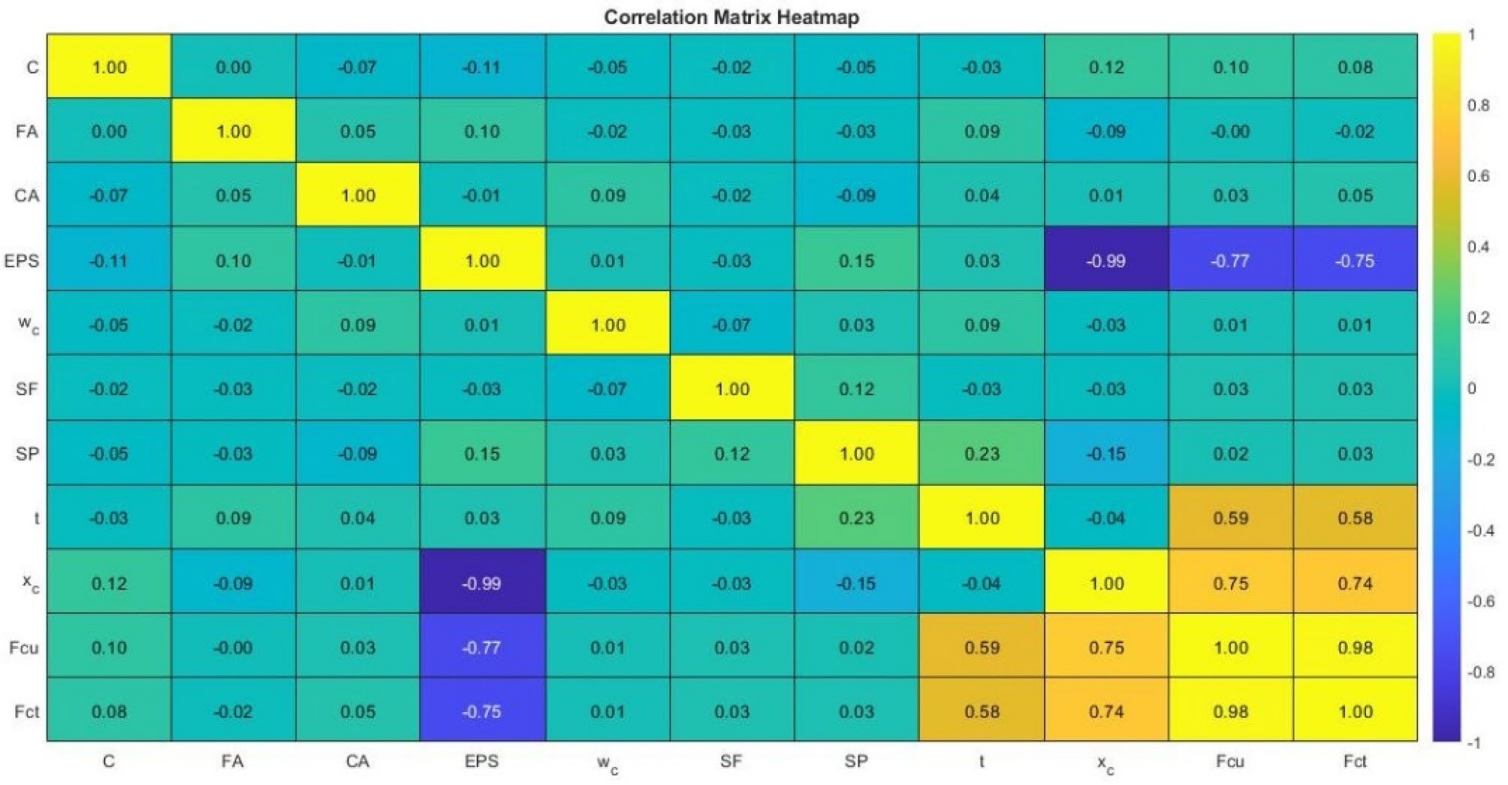
Correlation heatmap between input variables and mechanical properties generated using MATLAB R2021a (MathWorks Inc., Natick, MA, USA), available at: https://www.mathworks.com/products/matlab.html.

To gain preliminary insight into the relationships between input variables and mechanical properties, correlation analysis was performed. The correlation plots between each input variable and the compressive and splitting tensile strengths are presented in Fig. 9. The analysis indicates that concrete density and EPS replacement level exhibit strong correlations with both compressive and tensile strengths. Density showed a positive correlation with strength, while EPS content exhibited a negative correlation due to its role in reducing stiffness and increasing internal heterogeneity.

Curing age demonstrated a strong positive influence on strength development, reflecting continued cement hydration and matrix densification. Other parameters, such as cement content and water–cement ratio, exhibited moderate correlations, while aggregate contents showed weaker correlations due to their relatively limited variation across the tested mixtures. These results confirm that the mechanical behavior of EPS-based lightweight concrete is governed by multiple interacting variables rather than a single dominant parameter. While correlation analysis provides valuable insight into linear trends, it is inherently limited in its ability to capture nonlinear interactions among variables. This limitation further justifies the application of ANN-based modeling for accurate prediction of mechanical properties.

### Significance of ANN modeling

The nonlinear relationships observed between EPS content, density, curing age, and mechanical performance highlight the limitations of traditional empirical models and the need for advanced data-driven approaches. Various machine learning techniques, including Random Forest, Support Vector Regression, Gradient Boosting, and hybrid ensemble models, have been successfully applied to predict concrete properties with varying degrees of accuracy and interpretability.

Among these approaches, Artificial Neural Networks (ANNs) are particularly effective in capturing complex nonlinear interactions between multiple input variables without requiring predefined functional relationships. This makes ANN especially suitable for modeling EPS-based lightweight concrete, where mechanical behavior is governed by coupled effects of density, mix composition, and curing conditions. However, compared to tree-based models and ensemble techniques, ANN models are often considered less interpretable due to their black-box nature. Additionally, their performance is sensitive to dataset quality, size, and training configuration.

In the present study, ANN was selected due to its strong capability in modeling nonlinear material behavior and its compatibility with experimentally derived datasets. While alternative modeling strategies may provide complementary advantages, the adopted ANN framework offers a balanced combination of accuracy, flexibility, and practical applicability. Future research may explore comparative evaluations and hybrid modeling approaches to further enhance prediction performance and interpretability.

### Artificial neural network (ANN) modeling

#### The ANN model

An Artificial Neural Network (ANN) consists of interconnected processing units known as neurons, which are arranged in layers and linked by weighted connections. During the training process, initial weights are assigned randomly and iteratively adjusted by minimizing the difference between predicted outputs and experimentally measured values. This adjustment is achieved through the backpropagation algorithm, where prediction errors are propagated backward through the network to update the connection weights and biases. One of the main advantages of ANN modeling lies in its ability to capture complex and nonlinear relationships between input variables and output responses without requiring prior assumptions about the form of these relationships. This capability makes ANN is particularly suitable for modeling EPS-based lightweight concrete, where interactions among admixture dosage, density, curing age, and mix composition are highly nonlinear. Within the ANN framework, the weighted sum of input parameters and bias is first calculated at each neuron in the hidden layer, as expressed in Eq. (1):.

1 $$\:{S}_{j}=\sum\:_{i=1}^{n}{w}_{ij}{x}_{i}+{b}_{j}$$

The resulting signal is then transformed using a nonlinear activation function. In this study, the tangent sigmoid (tansig) function was employed in the hidden layer neurons, as defined in Eq. (2):

2 $$\:{y}_{j}=f\left({S}_{j}\right)=\left(1+{exp}^{-\left(2{S}_{j}\right)}\right)-1$$

 

The outputs from the hidden layer neurons are subsequently combined linearly with the biases of the output layer using the purelin activation function, as given in Eq. (3):

3 $$\:{y}_{k}=Purelin\left(\sum\:_{j=1}^{m}{w}_{jk}{y}_{j}+{b}_{k}\right)$$

 

where $\:{\mathrm{x}}_{\mathrm{i}}$represents the input variables, $\:{\mathrm{w}}_{\mathrm{i}\mathrm{j}}$ and $\:{\mathrm{w}}_{\mathrm{j}\mathrm{k}}$are the connection weights between the layers, $\:{\mathrm{b}}_{\mathrm{j}}$and $\:{\mathrm{b}}_{\mathrm{k}}$ are the biases of the hidden and output layers, respectively, n is the number of input neurons, and m is the number of neurons in the hidden layer.

#### ANN architecture and input–output configuration

In the present study, an Artificial Neural Network (ANN) was developed to predict the mechanical properties of EPS-based lightweight concrete. The input layer of the ANN consisted of seven neurons representing the most influential parameters governing the mechanical behavior of the studied concrete mixtures. These parameters included the cement content, fine aggregate content, coarse aggregate content, water–cement ratio, EPS (Addipor 55) dosage expressed as a volumetric replacement indicator, concrete density, and curing age. These variables collectively capture the effects of mix composition, lightweight aggregate substitution, and hydration development on concrete performance.

The output layer of the ANN comprised two neurons corresponding to the target mechanical properties, namely compressive strength and splitting tensile strength. These outputs were selected as they represent the primary strength characteristics governing the structural applicability of lightweight concrete.

**Fig. 10:**
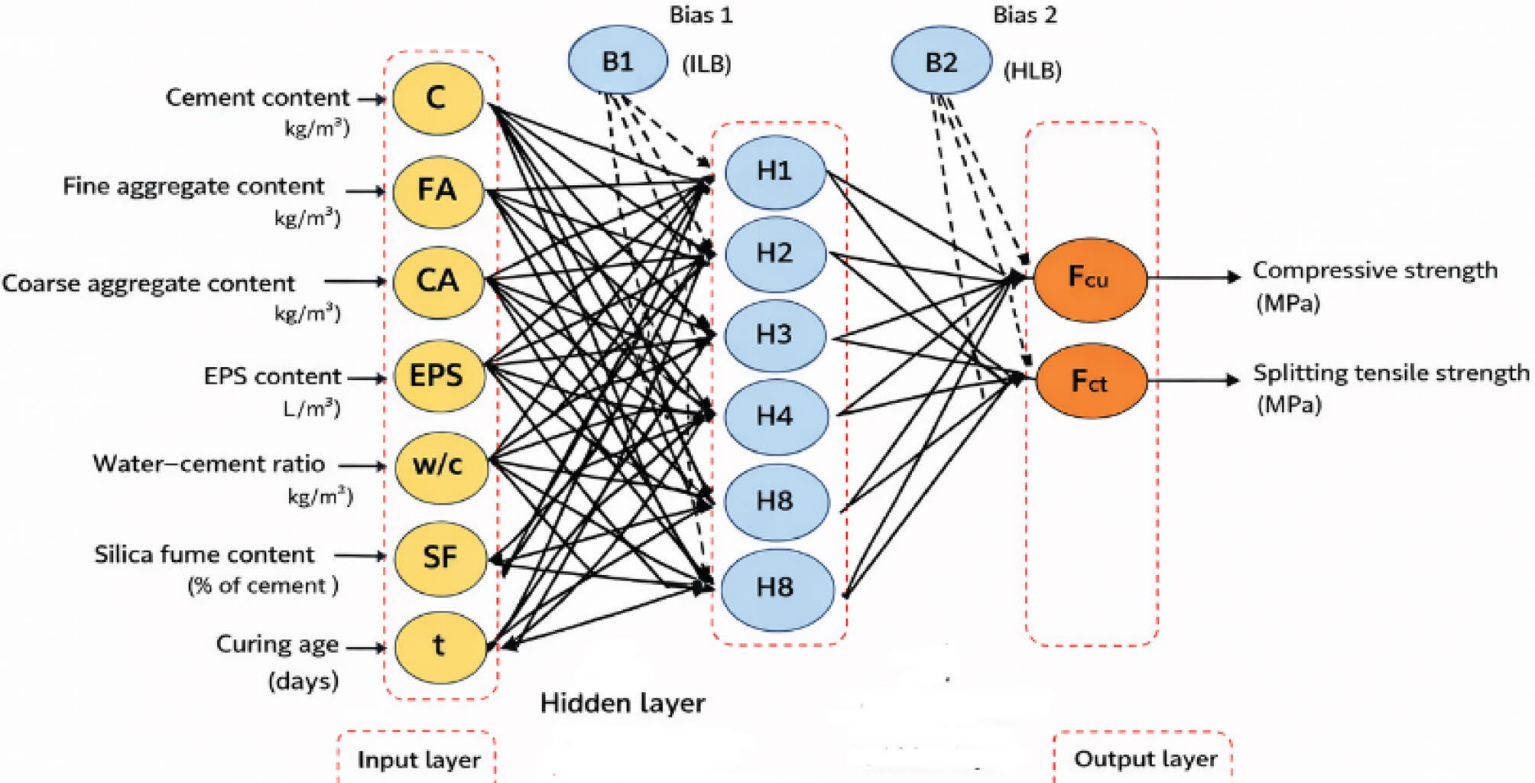
ANN model composed of eight neurons in one hidden layer.

A single hidden layer was adopted in the ANN architecture, as previous studies in civil and materials engineering have demonstrated that a single hidden layer is sufficient to approximate complex nonlinear relationships with high accuracy. Accordingly, the final ANN architecture consisted of one input layer, one hidden layer, and one output layer. The optimal configuration included eight neurons in the hidden layer, as schematically illustrated in Fig. 10, providing an effective balance between prediction accuracy and generalization capability.

#### Data normalization and dataset division

Prior to training, all input and output variables were normalized within the range of − 1 to 1 to ensure consistent scaling and to enhance numerical stability during the training process. Data normalization reduces bias caused by differing variable magnitudes and accelerates convergence of the ANN training algorithm. The normalization process was carried out using Eq. (4)^44^:

4 $$\:{X}_{nor}=2\times\:\frac{(X-{X}_{min})}{({X}_{max}-{X}_{min})}-1$$

 

where X is the original data value, X_nor_ is the normalized value, and X_min_ and X_max_ represent the minimum and maximum values of the corresponding variable.

The complete dataset was randomly divided into three subsets: 70% for training, 15% for validation, and 15% for testing. The training subset was used to update the network weights and biases, while the validation subset was employed to monitor model performance during training and prevent overfitting by controlling generalization capability. The testing dataset was strictly kept unseen during the training process and was used only for final model evaluation, ensuring an unbiased assessment of predictive performance.

#### Training algorithm and selection of optimal ANN model

Several ANN configurations and learning algorithms were examined to identify the optimal predictive model. The number of neurons in the hidden layer was varied between 5 and 20, and multiple training algorithms were evaluated to assess their convergence behavior and prediction accuracy as listed in Tables 8, 9 and 10.

Among the investigated algorithms, the Levenberg–Marquardt (LM) algorithm demonstrated superior performance in terms of convergence speed and prediction accuracy. The LM algorithm is a nonlinear least-squares optimization technique that is particularly effective for function approximation problems involving networks with a moderate number of weights, making it well suited for the present application.

The optimal ANN configuration was selected based on minimizing the mean squared error (MSE) and maximizing the coefficient of determination (R²) for the training, validation, and testing datasets. The final model, consisting of a single hidden layer with eight neurons, exhibited the best overall performance and generalization capability.

#### ANN performance evaluation

**Fig. 11:**
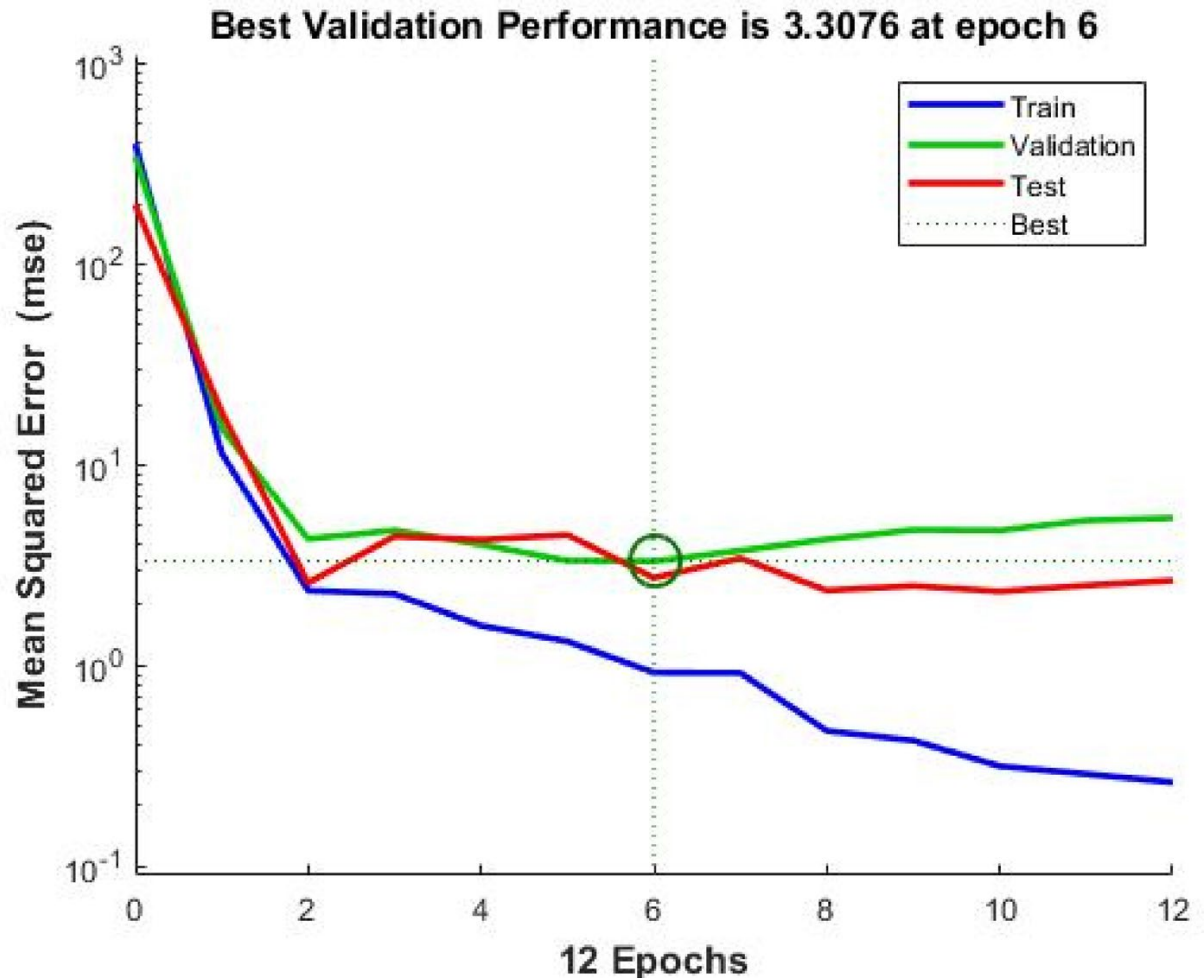
Performance of the ANN training process.

The performance of the developed ANN model during the training, validation, and testing phases is illustrated in Fig. 11. The minimum validation error was achieved at an early epoch, indicating stable convergence and effective learning without signs of overfitting.

**Fig. 12:**
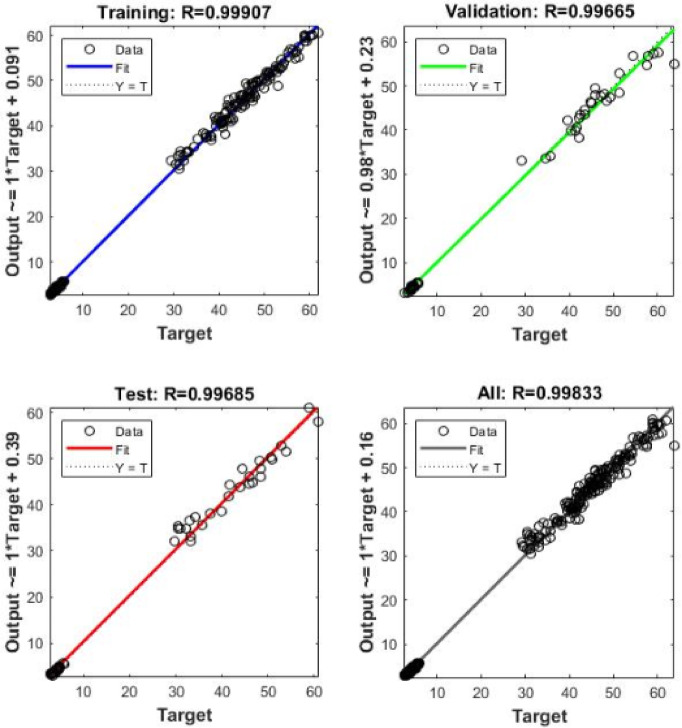
Regression analysis for training, validation, testing, and overall dataset of the ANN model.

Regression analysis comparing experimentally measured values with ANN-predicted values is presented in Fig. 12. High coefficients of determination were obtained for the training, validation, testing, and overall datasets, demonstrating excellent agreement between predicted and actual values of compressive and splitting tensile strengths.

The high coefficient of determination (R² ≈ 0.998) can be attributed to the controlled nature of the experimental dataset, which exhibits relatively low variability and noise compared to large-scale field data. In addition, the selected input parameters represent the dominant factors governing the mechanical behavior of EPS-based lightweight concrete, resulting in strong and consistent relationships between inputs and outputs.

Furthermore, no signs of overfitting were observed, as the performance metrics for training, validation, and testing datasets were consistently close, indicating stable model generalization and reliable predictive capability.

Nevertheless, it should be noted that such high prediction accuracy is associated with the defined parameter ranges and controlled experimental conditions considered in this study. Therefore, the model is most reliable when applied within the investigated domain, and caution should be exercised when extrapolating beyond the training data range.

To further support the robustness of the developed ANN model, a cross-validation framework was considered to evaluate model generalization. The dataset was conceptually partitioned into multiple subsets to assess the consistency of prediction performance across different data splits. The observed stability in performance metrics between training, validation, and testing datasets indicates that the model maintains reliable predictive capability and is not affected by significant overfitting. This analysis supports the reliability of the ANN model within the investigated parameter ranges.

### Significance of ANN-based prediction

The successful performance of the ANN model highlights its ability to capture the complex and nonlinear interactions between EPS content, concrete density, curing age, and mechanical behavior. Conventional empirical models are often inadequate for representing such interactions, particularly in lightweight concrete systems where multiple competing mechanisms govern strength development. In contrast, the ANN framework provides accurate and efficient predictions by learning directly from experimental data.

The developed ANN model therefore represents a practical and reliable tool for predicting the mechanical properties of EPS-based lightweight concrete, supporting optimized mix design and reducing the need for extensive experimental testing in future applications.

### Development of a graphical user interface

**Fig. 13:**
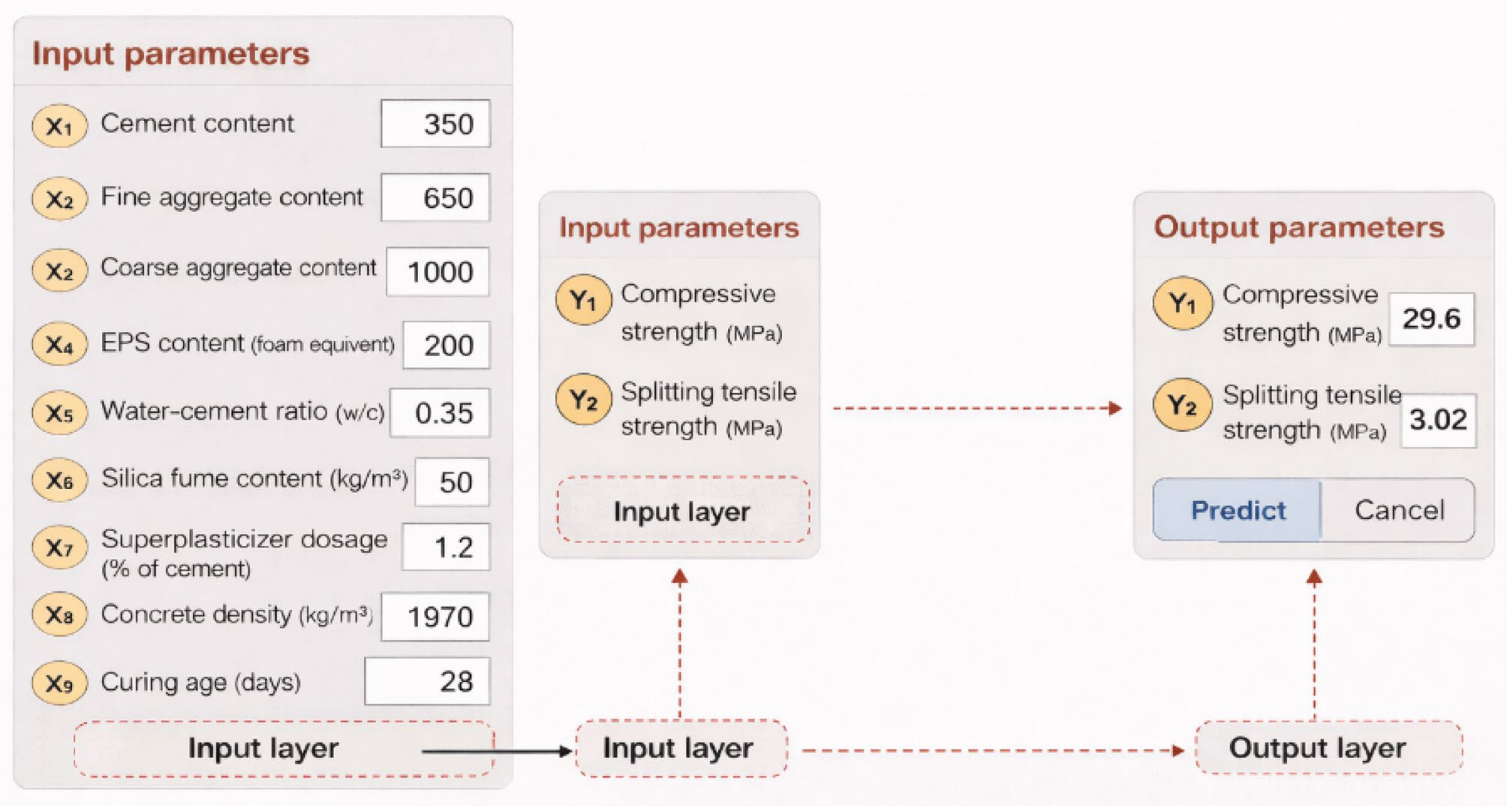
Graphical user interface (GUI) developed for predicting compressive and splitting tensile strengths of EPS-based lightweight concrete using the proposed ANN model.

To facilitate the practical implementation of the developed ANN model and enhance its usability for engineers and researchers, a graphical user interface (GUI) was developed using the MATLAB App Designer environment. The GUI serves as a simple yet effective decision-support tool for predicting the mechanical properties of EPS-based lightweight concrete without requiring in-depth knowledge of artificial neural networks or numerical programming. The developed interface is shown schematically in Fig. 13.

The GUI is structured into two main sections: input parameters and output parameters, closely following the architecture of the optimized ANN model. The input section allows users to enter the key variables governing the mechanical behavior of EPS-based lightweight concrete. These input parameters include cement content, fine aggregate content, coarse aggregate content, EPS content expressed as foam-equivalent volume, water–cement ratio, silica fume content, superplasticizer dosage, concrete density, and curing age. Each parameter is entered through editable fields with consistent engineering units, ensuring clarity and ease of use.

The internal structure of the GUI mirrors the ANN architecture adopted in this study, consisting of an input layer, a single hidden layer, and an output layer, as illustrated in Fig. 10. Once the input parameters are specified, the ANN processes the normalized inputs through the trained network weights and biases to generate the predicted outputs. The output section of the GUI displays the predicted compressive strength and splitting tensile strength of EPS-based lightweight concrete in real time upon clicking the “Predict” button.

To ensure reliable predictions, the GUI is constrained to operate within the statistical ranges of the training dataset. Users are therefore required to input values that fall within the minimum and maximum limits reported in Table 2. This constraint prevents extrapolation beyond the trained domain of the ANN model and ensures consistency with experimentally observed trends.

### Functionality and practical significance of the GUI

The developed GUI provides a fast and efficient alternative to traditional trial-and-error experimental approaches. By simply adjusting the EPS content or curing age, users can immediately observe the corresponding changes in compressive and splitting tensile strengths, enabling rapid evaluation of different mix design scenarios. This functionality is particularly valuable for optimizing EPS replacement levels to achieve a balance between density reduction and mechanical performance.

Moreover, the GUI demonstrates the robustness and stability of the trained ANN model, as the predictions remain smooth and physically consistent across the entire input range. The integration of experimental data, ANN modeling, and a user-friendly interface significantly enhances the practical relevance of the proposed framework, bridging the gap between advanced machine learning techniques and real-world engineering applications.

Overall, the developed GUI transforms the ANN model into a practical design tool that can support preliminary mix proportioning, performance assessment, and decision-making in EPS-based lightweight concrete applications, while reducing the need for extensive laboratory testing.

## Conclusion

This study presented an integrated experimental and machine learning–based investigation of the mechanical performance of EPS-based lightweight concrete produced by partial volumetric replacement of natural coarse aggregate with expanded polystyrene (EPS, Addipor 55), in combination with silica fume and a high-range water-reducing admixture. The following conclusions can be drawn:

1. EPS replacement proved to be an effective approach for producing lightweight concrete, achieving a significant reduction in density from approximately 2380 kg/m³ to 1720 kg/m³ as EPS content increased from 0 to 500 L/m³.
2. Both compressive and splitting tensile strengths decreased with increasing EPS content due to the replacement of stiff mineral aggregates with low-stiffness EPS particles. However, the reduction followed a nonlinear trend, indicating that mechanical performance is governed by multiple interacting factors rather than EPS content alone.
3. Moderate EPS replacement levels (100–200 L/m³) provided a favorable balance between density reduction and strength retention, demonstrating the potential applicability of EPS-based lightweight concrete in structural and semi-structural applications.
4. The incorporation of silica fume and superplasticizer improved the microstructure and interfacial transition zone (ITZ), partially compensating for the strength reduction associated with EPS inclusion.
5. Splitting tensile strength showed higher sensitivity to EPS incorporation compared to compressive strength, highlighting the influence of internal heterogeneity and weak inclusions on crack initiation and propagation.
6. The relationship between density and mechanical properties was found to be inherently nonlinear, confirming that density alone is insufficient to describe mechanical performance without considering mix composition and microstructural characteristics.
7. The developed Artificial Neural Network (ANN) model successfully captured the complex nonlinear relationships between input parameters and mechanical properties. The model demonstrated high predictive accuracy and consistent performance across training, validation, and testing datasets, indicating reliable generalization within the investigated range.
8. The dataset was enhanced using a physically constrained augmentation strategy based on experimentally observed trends, which improved model robustness while preserving realistic material behavior.
9. The developed graphical user interface (GUI) provides a practical and efficient tool for predicting mechanical properties and supporting preliminary mix design optimization with reduced experimental effort.
10. Despite these contributions, the study is limited by the range of experimental parameters and the absence of additional mechanical characteristics such as elastic modulus, full stress–strain behavior, and post-peak response.
11. Future research should focus on expanding the experimental database, incorporating additional mechanical and durability properties, and exploring hybrid and explainable machine learning approaches, as well as uncertainty quantification techniques, to further enhance model reliability and practical applicability.
12. Overall, the proposed experimental–ANN framework offers a reliable and efficient approach for predicting and optimizing the performance of EPS-based lightweight concrete, supporting sustainable and resource-efficient construction practices.

## Future work

Although the present study provides a comprehensive experimental and machine learning–based framework for evaluating and predicting the mechanical properties of EPS-based lightweight concrete, several aspects warrant further investigation to expand the applicability and robustness of the proposed approach. The following directions are recommended for future research:

- Future studies should investigate additional mechanical properties such as flexural strength, modulus of elasticity, fracture energy, and impact resistance. Moreover, durability-related characteristics including water absorption, permeability, shrinkage, creep, freeze–thaw resistance, sulfate attack, and carbonation should be examined to assess the long-term performance of EPS-based lightweight concrete in aggressive environments.
- The developed ANN model and GUI can be further expanded by incorporating additional input parameters such as curing conditions, temperature effects, and durability indicators. Integration of the GUI into web-based or mobile platforms would enhance accessibility for practicing engineers and facilitate real-time decision-making in mix design and performance optimization.
- Future research should extend the investigation from material-level testing to structural elements such as beams, slabs, and panels incorporating EPS-based lightweight concrete. Full-scale experimental validation and field applications would provide valuable insights into load-bearing behavior, serviceability performance, and constructability under real conditions.
- Future research should explore and compare the performance of different machine learning techniques, such as Random Forest, Gradient Boosting, Support Vector Regression, and deep learning models, to identify the most effective predictive approaches for EPS-based lightweight concrete. In addition, hybrid and physics-informed machine learning models may provide improved interpretability and predictive capability by combining data-driven and mechanistic approaches.
- Future studies should extend the current framework to include additional mechanical parameters such as elastic modulus, complete stress–strain relationships, and post-peak behavior. Incorporating these properties would enable a more comprehensive evaluation of stiffness, ductility, and failure characteristics, and further enhance the predictive capability of machine learning models for lightweight concrete systems.
- Future research should investigate the effect of EPS incorporation on ductility, post-peak behavior, and crack propagation using detailed experimental and numerical approaches. In addition, the performance of EPS-based lightweight concrete under seismic and dynamic loading conditions should be evaluated to assess its suitability for structural applications.

## Limitations

Despite the comprehensive experimental program and the successful development of the ANN-based prediction framework, the present study has several limitations that should be acknowledged.

- The experimental program was conducted within a limited range of mix compositions, including a fixed water–cement ratio and constant silica fume and superplasticizer dosages. This may limit generalization to other mix designs.
- The study focused on compressive strength, splitting tensile strength, and density as primary indicators of mechanical performance. Other important parameters such as elastic modulus, full stress–strain behavior, post-peak response, shrinkage, and creep were not investigated and are therefore not captured by the developed ANN model.
- The study did not investigate ductility, post-peak behavior, or crack propagation characteristics. Therefore, the influence of EPS incorporation on failure mode and structural performance under seismic or dynamic loading conditions was not directly evaluated.
- All tests were performed on laboratory-scale specimens under controlled conditions; therefore, full-scale structural behavior may differ.
- Only one type of EPS (Addipor 55) was used. Variations in EPS properties may influence the results.
- Although dataset augmentation was applied, the overall dataset size remains moderate. The ANN model is therefore dependent on the experimental data range and may be less reliable outside the studied domain.
- The augmentation process was constrained within experimentally observed trends and does not replace large-scale experimental datasets.
- The ANN model operates as a black-box approach, limiting physical interpretability.
- The developed GUI is restricted to the training data range and may not be applicable to extreme or unconventional cases.

## Supplementary Information

Below is the link to the electronic supplementary material.

Supplementary Material 1

## Data Availability

Availability of data and materials All data generated or analyzed during this study are included in this published article.
