# Supplementary material for: Machine learning–driven prediction of mechanical properties of lightweight concrete based on experimental data
Source: Sci Rep. 2026 Jun 3;16:17187. doi: 10.1038/s41598-026-55467-z (PMC13234110; doi:10.1038/s41598-026-55467-z)
Supplement: Supplementary file 1 — Supplementary Material 1 [file 41598_2026_55467_MOESM1_ESM.docx]

Table S1. Chemical composition of Ordinary Portland Cement (OPC).

| **Oxide** | **OPC Content (%)** |
| --- | --- |
| SiO₂ (Silicon Dioxide) | 21.30 |
| Al₂O₃ (Aluminum Oxide) | 5.40 |
| Fe₂O₃ (Ferric Oxide) | 3.20 |
| CaO (Calcium Oxide) | 63.10 |
| MgO (Magnesium Oxide) | 2.40 |
| K₂O (Potassium Oxide) | 0.60 |
| Na₂O (Sodium Oxide) | 0.20 |
| SO₃ (Sulfur Trioxide) | 2.80 |
| TiO₂ (Titanium Dioxide) | 0.20 |
| P₂O₅ (Phosphorus Pentoxide) | 0.10 |
| MnO (Manganese Oxide) | 0.05 |
| Cl (Chloride) | 0.02 |
| Loss on Ignition (LOI) | 1.00 |

**Table S2. Physical properties of aggregates.**

| **Property** | **Fine aggregate (Sand)** | **Coarse aggregate** |
| --- | --- | --- |
| Maximum size (mm) | 4.75 | 20 |
| Fineness modulus | 2.6–2.8 | — |
| Specific gravity | 2.60 | 2.65 |
| Water absorption (%) | 1.5 | 0.8 |
| Bulk density (kg/m³) | 1600 | 1500 |

**Table S3. Physical properties of expanded polystyrene (EPS – Addipor 55)**

| **Property** | **Value** | **Test method / Source** |
| --- | --- | --- |
| Material type | Expanded polystyrene (EPS) granules | Manufacturer datasheet |
| Particle shape | Rounded / irregular beads | Visual inspection |
| Particle size range (mm) | 2–6 | Manufacturer datasheet |
| Bulk density (kg/m³) | 15–25 | ASTM C29 (adapted) |
| Specific gravity | 0.02–0.03 | Calculated |
| Water absorption (%) | < 1.0 | ASTM C128 (modified) |
| Thermal conductivity (W/m·K) | 0.035–0.040 | Manufacturer datasheet |
| Compressive strength at 10% strain (kPa) | 100–150 | ASTM D1621 |
| Chemical reactivity | Inert | Literature |
| Durability | Non-biodegradable, chemically stable | Literature |

**Note:** Addipor 55 is an EPS-based lightweight aggregate used as a volumetric replacement of natural coarse aggregate. It is chemically inert and does not participate in cement hydration. Its primary role is to reduce concrete density while maintaining acceptable mechanical performance.

**Table S4:** Compressive strength results of EPS-based lightweight concrete

| **Mix ID** | **EPS (Foam equivalent, L/m³)** | **3 Days (MPa)** | **7 Days (MPa)** | **28 Days (MPa)** |
| --- | --- | --- | --- | --- |
| C0 | 0 | 23.5 | 32.0 | 39.0 |
| C-SF-0 | 0 | 24.8 | 33.5 | 41.2 |
| C-SF-10 | 100 | 23.6 | 31.8 | 38.0 |
| C-SF-20 | 200 | 22.4 | 30.0 | 35.8 |
| C-SF-30 | 300 | 21.0 | 28.2 | 33.5 |
| C-SF-40 | 400 | 19.6 | 26.5 | 31.0 |
| C-SF-50 | 500 | 18.0 | 24.5 | 28.6 |

**Table S5:** Splitting tensile strength results of EPS-based lightweight concrete

| **Mix ID** | **EPS (Foam equivalent, L/m³)** | **3 Days (MPa)** | **7 Days (MPa)** | **28 Days (MPa)** |
| --- | --- | --- | --- | --- |
| C0 | 0 | 2.45 | 3.20 | 4.00 |
| C-SF-0 | 0 | 2.70 | 3.45 | 4.30 |
| C-SF-10 | 100 | 2.55 | 3.20 | 3.85 |
| C-SF-20 | 200 | 2.40 | 3.00 | 3.55 |
| C-SF-30 | 300 | 2.20 | 2.75 | 3.25 |
| C-SF-40 | 400 | 1.95 | 2.55 | 2.95 |
| C-SF-50 | 500 | 1.75 | 2.30 | 2.65 |

**Table S6:** Density results of EPS-based lightweight concrete mixtures

| **Mix ID** | **EPS (Foam equivalent, L/m³)** | **Density (kg/m³)** |
| --- | --- | --- |
| C0 | 0 | 2380 |
| C-SF-0 | 0 | 2350 |
| C-SF-10 | 100 | 2200 |
| C-SF-20 | 200 | 2100 |
| C-SF-30 | 300 | 1980 |
| C-SF-40 | 400 | 1850 |
| C-SF-50 | 500 | 1720 |

## ****Table S7. Datasets****

##
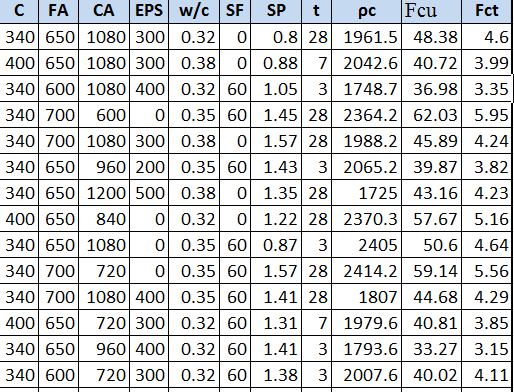


## ****Table S8. Training performance of ANN models (MSE)****

| **Neus.** | **1st** | **2nd** | **3rd** | **4th** | **5th** | **6th** | **7th** | **8th** | **9th** | **10th** | **11th** |
| --- | --- | --- | --- | --- | --- | --- | --- | --- | --- | --- | --- |
| 5 | 6.20E-04 | 8.10E-04 | 1.45E-03 | 1.30E-03 | 1.55E-03 | 9.80E-04 | 1.05E-03 | 9.70E-04 | 1.32E-03 | 3.80E-01 | 2.10E-01 |
| 6 | 5.40E-04 | 7.50E-04 | 1.10E-03 | 2.80E-03 | 1.05E-03 | 9.20E-04 | 1.30E-03 | 2.95E-03 | 4.80E-03 | 4.20E-01 | 1.40E-01 |
| 7 | 4.30E-04 | 2.10E-03 | 1.55E-03 | 9.40E-04 | 1.25E-03 | 1.10E-03 | 1.20E-03 | 8.40E-04 | 2.20E-03 | 1.10E-01 | 1.95E+00 |
| **8** | **6.10E-05** | 7.40E-04 | 8.90E-04 | 7.90E-04 | 1.10E-03 | 1.25E-03 | 2.10E-03 | 2.35E-03 | 2.05E-03 | 4.10E-01 | 3.05E-01 |
| 9 | 5.90E-04 | 8.30E-04 | 7.40E-04 | 1.10E-03 | 6.80E-04 | 1.05E-03 | 1.25E-03 | 9.80E-04 | 1.30E-03 | 2.70E-01 | 4.80E-01 |
| 10 | 6.80E-04 | 9.10E-04 | 6.80E-04 | 1.20E-03 | 8.20E-04 | 8.10E-04 | 1.55E-03 | 1.45E-03 | 1.70E-03 | 9.80E-01 | 5.10E-01 |
| 11 | 5.10E-04 | 7.90E-04 | 7.20E-04 | 1.30E-03 | 9.50E-04 | 7.80E-04 | 1.35E-03 | 1.10E-03 | 1.60E-03 | 3.10E-01 | 9.50E-02 |
| 12 | 6.00E-04 | 9.60E-04 | 7.40E-04 | 1.20E-03 | 7.90E-04 | 8.20E-04 | 1.70E-03 | 1.00E-03 | 1.85E-03 | 4.50E-02 | 6.20E-02 |
| 13 | 3.80E-04 | 9.10E-04 | 1.50E-03 | 1.60E-03 | 8.20E-04 | 6.90E-04 | 1.30E-03 | 1.10E-03 | 2.20E-03 | 1.90E-01 | 1.70E-01 |
| 14 | 5.60E-04 | 1.05E-03 | 7.80E-04 | 1.00E-03 | 6.50E-04 | 7.90E-04 | 3.40E-03 | 2.40E-03 | 3.10E-03 | 6.90E-01 | 2.80E-02 |
| 15 | 4.10E-04 | 7.90E-04 | 2.10E-03 | 2.30E-03 | 7.80E-04 | 9.90E-04 | 8.60E-04 | 3.00E-03 | 2.40E-03 | 2.70E-01 | 3.40E-01 |
| 16 | 4.70E-04 | 9.20E-04 | 2.90E-03 | 1.10E-03 | 7.50E-04 | 1.25E-03 | 1.20E-03 | 9.10E-04 | 1.55E-02 | 1.00E-01 | 9.80E-02 |
| 17 | 4.30E-04 | 7.10E-04 | 6.50E-04 | 1.45E-03 | 9.90E-04 | 7.80E-04 | 1.10E-03 | 2.10E-03 | 3.60E-02 | 8.20E-01 | 2.60E-01 |
| 18 | 5.30E-04 | 9.80E-04 | 1.40E-03 | 8.50E-04 | 1.00E-03 | 6.30E-04 | 6.70E-04 | 2.40E-03 | 2.30E-03 | 1.60E-01 | 1.70E+00 |
| 19 | 4.30E-04 | 7.10E-04 | 1.10E-03 | 1.10E-03 | 7.10E-04 | 6.60E-04 |  |  |  |  |  |

# ****Table S9. Validation performance of ANN models (MSE)****

| **Neus.** | **1st** | **2nd** | **3rd** | **4th** | **5th** | **6th** | **7th** | **8th** | **9th** | **10th** | **11th** |
| --- | --- | --- | --- | --- | --- | --- | --- | --- | --- | --- | --- |
| 5 | 7.40E-04 | 8.60E-04 | 1.60E-03 | 1.30E-03 | 1.10E-03 | 8.70E-04 | 1.10E-03 | 1.30E-03 | 1.55E-03 | 3.95E-01 | 2.00E-01 |
| 6 | 5.30E-04 | 9.60E-04 | 1.30E-03 | 3.20E-03 | 1.25E-03 | 8.10E-04 | 1.25E-03 | 1.55E-03 | 3.80E-03 | 4.20E-01 | 1.55E-01 |
| 7 | 4.20E-04 | 1.80E-03 | 2.00E-03 | 5.50E-04 | 8.50E-04 | 9.60E-04 | 1.40E-03 | 7.10E-04 | 3.20E-03 | 1.25E-01 | 2.20E+00 |
| **8** | **8.90E-05** | 6.90E-04 | 1.10E-03 | 9.50E-04 | 1.10E-03 | 1.95E-03 | 1.90E-03 | 2.55E-03 | 3.30E-03 | 4.30E-01 | 3.30E-01 |
| 9 | 8.80E-04 | 1.70E-03 | 9.50E-04 | 7.50E-04 | 6.80E-04 | 9.10E-04 | 1.15E-03 | 1.90E-03 | 1.30E-03 | 3.50E-01 | 5.00E-01 |
| 10 | 7.30E-04 | 2.10E-03 | 8.50E-04 | 7.80E-04 | 8.40E-04 | 1.15E-03 | 7.90E-04 | 1.10E-03 | 1.85E-03 | 1.10E+00 | 5.40E-01 |
| … | … | … | … | … | … | … | … | … | … | … | … |

## ****Table S10. Testing performance of ANN models (MSE)****

| **Neus.** | **1st** | **2nd** | **3rd** | **4th** | **5th** | **6th** | **7th** | **8th** | **9th** | **10th** | **11th** |
| --- | --- | --- | --- | --- | --- | --- | --- | --- | --- | --- | --- |
| 5 | 8.20E-04 | 7.60E-04 | 9.30E-04 | 2.50E-03 | 9.70E-04 | 8.10E-04 | 9.60E-04 | 1.30E-03 | 2.30E-03 | 4.30E-01 | 1.95E-01 |
| 6 | 7.10E-04 | 8.00E-04 | 1.45E-03 | 3.20E-03 | 8.80E-04 | 1.35E-03 | 1.45E-03 | 5.90E-03 | 5.00E-03 | 4.50E-01 | 1.45E-01 |
| 7 | 5.00E-04 | 2.40E-03 | 2.60E-03 | 7.00E-04 | 1.45E-03 | 1.25E-03 | 2.00E-03 | 1.35E-03 | 7.40E-03 | 1.35E-01 | 2.25E+00 |
| **8** | **1.10E-04** | 1.40E-03 | 6.30E-04 | 6.50E-04 | 2.35E-03 | 9.50E-04 | 2.15E-03 | 3.15E-03 | 2.80E-03 | 4.35E-01 | 3.55E-01 |
| 9 | 7.50E-04 | 9.00E-04 | 6.80E-04 | 1.15E-03 | 7.10E-04 | 1.15E-03 | 2.15E-03 | 8.50E-04 | 1.45E-03 | 3.70E-01 | 5.10E-01 |
| 10 | 1.05E-03 | 2.20E-03 | 5.10E-04 | 1.10E-03 | 6.50E-04 | 6.80E-04 | 2.70E-03 | 2.40E-03 | 2.10E-03 | 1.15E+00 | 5.60E-01 |
| 11 | 4.90E-04 | 6.40E-04 | 7.20E-04 | 1.65E-03 | 1.75E-03 | 9.90E-04 | 1.05E-03 | 1.70E-03 | 2.85E-03 | 2.90E-01 | 7.20E-02 |
| 12 | 6.90E-04 | 1.15E-03 | 1.45E-03 | 1.20E-03 | 7.40E-04 | 3.35E-03 | 8.50E-04 | 1.50E-03 | 2.35E-03 | 3.90E-02 | 3.80E-02 |
| 13 | 3.90E-04 | 9.50E-04 | 1.70E-03 | 1.60E-03 | 6.70E-04 | 8.90E-04 | 1.50E-03 | 2.10E-03 | 1.75E-03 | 1.65E-01 | 1.45E-01 |
| 14 | 7.60E-04 | 1.05E-03 | 9.60E-04 | 1.75E-03 | 1.20E-03 | 6.50E-04 | 5.60E-03 | 3.20E-03 | 2.10E-03 | 7.20E-01 | 2.20E-02 |
| 15 | 4.80E-04 | 8.20E-04 | 2.10E-03 | 2.20E-03 | 1.30E-03 | 8.40E-04 | 1.70E-03 | 1.70E-03 | 2.00E-03 | 2.30E-01 | 3.30E-01 |
| 16 | 4.60E-04 | 1.00E-03 | 3.00E-03 | 1.90E-03 | 9.10E-04 | 1.40E-03 | 1.25E-03 | 7.80E-04 | 1.80E-02 | 9.50E-02 | 1.05E-01 |
| 17 | 7.30E-04 | 2.55E-03 | 1.15E-03 | 1.50E-03 | 2.15E-03 | 9.10E-04 | 1.55E-03 | 2.00E-03 | 4.10E-02 | 8.30E-01 | 2.45E-01 |
| 18 | 2.60E-03 | 1.20E-03 | 1.80E-03 | 8.70E-04 | 1.30E-03 | 7.10E-04 | 7.40E-04 | 2.60E-03 | 3.20E-03 | 1.50E-01 | 1.70E+00 |
| 19 | 6.60E-04 | 9.90E-04 | 1.75E-03 | 1.35E-03 | 1.20E-03 | 6.70E-04 | 3.10E-03 | 1.30E-03 | 3.40E-02 | 8.40E-02 | 5.20E+00 |
| 20 | 3.20E-03 | 1.45E-03 | 4.40E-03 | 2.45E-03 | 1.45E-03 | 2.35E-03 | 1.20E-03 | 2.60E-03 | 1.70E-02 | 1.00E+00 | 4.00E-01 |
